# Supplementary material for: Molecular diversity of Uzbekistan’s fishes assessed with DNA barcoding
Source: Sci Rep. 2021 Aug 19;11:16894. doi: 10.1038/s41598-021-96487-1 (PMC8376971; doi:10.1038/s41598-021-96487-1)
Supplement: Supplementary file 2 — Supplementary Information 2. [file 41598_2021_96487_MOESM2_ESM.pdf]

**Title**	**Molecular diversity of Uzbekistan's fishes assessed with DNA barcoding**
Authors	Bakhtiyor SHERALIEV[1,2], Zuogang PENG[1]
Affiliation	[1]Key Laboratory of Freshwater Fish Reproduction and Development (Ministry of Education), Southwest University, School of Life Sciences, Chongqing 400715, China
	[2]Faculty of Life Sciences, Fergana State University, Fergana, Uzbekistan
*Corresponding author	Professor Zuogang PENG, Ph.D.<br>E-mail: pzg@swu.edu.cn

TABLE S1. Voucher metadata.

Identifications and metadata are subject to revision and may change

No.	Sample ID	Voucher number	BOLD process ID	GenBank Acc. No.	Family	Species	Collected by	Identified by	Region	Drainage	River/stream/ hill/lake	Latitude	Longitude	Date
1.	Rhodeus_ocellatus_261	SWU19072019261	FFU001-20	MW649153	Acheilognathidae	*Rhodeus ocellatus*	Sheraliev, BM	Sheraliev, BM	Surxondaryo	Amu Darya	Surkhan Darya	37.255833	67.338611	19/07/2019
2.	Rhodeus_ocellatus_284	SWU20072019284	FFU002-20	MW649154	Acheilognathidae	*Rhodeus ocellatus*	Sheraliev, BM	Sheraliev, BM	Surxondaryo	Amu Darya	Amu Darya	37.235555	67.660555	20/07/2019
3.	Rhodeus_ocellatus_353	SWU22072019353	FFU003-20	MW649155	Acheilognathidae	*Rhodeus ocellatus*	Sheraliev, BM	Sheraliev, BM	Surxondaryo	Amu Darya	Karatag river	38.363055	68.072059	22/07/2019
4.	Rhodeus_ocellatus_354	SWU22072019354	FFU004-20	MW649156	Acheilognathidae	*Rhodeus ocellatus*	Sheraliev, BM	Sheraliev, BM	Surxondaryo	Amu Darya	Karatag river	38.363055	68.072059	22/07/2019
5.	Rhodeus_ocellatus_355	SWU22072019355	FFU005-20	MW649157	Acheilognathidae	*Rhodeus ocellatus*	Sheraliev, BM	Sheraliev, BM	Surxondaryo	Amu Darya	Karatag river	38.363055	68.072059	22/07/2019
6.	Rhodeus_ocellatus_356	SWU22072019356	FFU006-20	MW649158	Acheilognathidae	*Rhodeus ocellatus*	Sheraliev, BM	Sheraliev, BM	Surxondaryo	Amu Darya	Karatag river	38.363055	68.072059	22/07/2019
7.	Rhodeus_ocellatus_357	SWU22072019357	FFU007-20	MW649159	Acheilognathidae	*Rhodeus ocellatus*	Sheraliev, BM	Sheraliev, BM	Surxondaryo	Amu Darya	Karatag river	38.363055	68.072059	22/07/2019
8.	Rhodeus_ocellatus_390	SWU24072019390	FFU008-20	MW649160	Acheilognathidae	*Rhodeus ocellatus*	Sheraliev, BM	Sheraliev, BM	Surxondaryo	Amu Darya	Surkhan Darya	37.823333	67.618611	24/07/2019
9.	Rhodeus_ocellatus_460	SWU28072019460	FFU009-20	MW649161	Acheilognathidae	*Rhodeus ocellatus*	Sheraliev, BM	Sheraliev, BM	Bukhara	Zeravshan	Zeravshan	40.071012	64.778429	28/07/2019
10.	Rhodeus_ocellatus_461	SWU28072019461	FFU010-20	MW649162	Acheilognathidae	*Rhodeus ocellatus*	Sheraliev, BM	Sheraliev, BM	Bukhara	Zeravshan	Zeravshan	40.071012	64.778429	28/07/2019
11.	Rhodeus_ocellatus_462	SWU28072019462	FFU011-20	MW649163	Acheilognathidae	*Rhodeus ocellatus*	Sheraliev, BM	Sheraliev, BM	Bukhara	Zeravshan	Zeravshan	40.071012	64.778429	28/07/2019
12.	Rhodeus_ocellatus_550	SWU09082019550	FFU012-20	MW649164	Acheilognathidae	*Rhodeus ocellatus*	Sheraliev, BM	Sheraliev, BM	Namangan	Syr Darya	Kara Darya	40.918813	71.828896	09/08/2019
13.	Rhodeus_ocellatus_570	SWU09082019570	FFU013-20	MW649165	Acheilognathidae	*Rhodeus ocellatus*	Sheraliev, BM	Sheraliev, BM	Namangan	Syr Darya	Kara Darya	40.918813	71.828896	09/08/2019
14.	Rhodeus_ocellatus_572	SWU09082019572	FFU014-20	MW649166	Acheilognathidae	*Rhodeus ocellatus*	Sheraliev, BM	Sheraliev, BM	Namangan	Syr Darya	Kara Darya	40.918813	71.828896	09/08/2019
15.	Rhodeus_ocellatus_573	SWU09082019573	FFU015-20	MW649167	Acheilognathidae	*Rhodeus ocellatus*	Sheraliev, BM	Sheraliev, BM	Namangan	Syr Darya	Kara Darya	40.918813	71.828896	09/08/2019
16.	Rhodeus_ocellatus_585	SWU09082019585	FFU016-20	MW649168	Acheilognathidae	*Rhodeus ocellatus*	Sheraliev, BM	Sheraliev, BM	Namangan	Syr Darya	Naryn River	40.940911	71.853631	09/08/2019
17.	Rhodeus_ocellatus_614	SWU18082019614	FFU017-20	MW649169	Acheilognathidae	*Rhodeus ocellatus*	Sheraliev, BM	Sheraliev, BM	Tashkent	Syr Darya	Chirchik River	41.274444	69.389166	18/08/2019
18.	Rhodeus_ocellatus_615	SWU18082019615	FFU018-20	MW649170	Acheilognathidae	*Rhodeus ocellatus*	Sheraliev, BM	Sheraliev, BM	Tashkent	Syr Darya	Chirchik River	41.274444	69.389166	18/08/2019
19.	Rhodeus_ocellatus_616	SWU18082019616	FFU019-20	MW649171	Acheilognathidae	*Rhodeus ocellatus*	Sheraliev, BM	Sheraliev, BM	Tashkent	Syr Darya	Chirchik River	41.274444	69.389166	18/08/2019
20.	Rhodeus_ocellatus_712	SWU16052020712	FFU020-20	MW649172	Acheilognathidae	*Rhodeus ocellatus*	Rozimov, A	Sheraliev, BM	Khorazm	Amu Darya	Unnamed stream	41.540222	60.166666	16/05/2020
21.	Rhodeus_ocellatus_713	SWU17052020713	FFU021-20	MW649173	Acheilognathidae	*Rhodeus ocellatus*	Rozimov, A	Sheraliev, BM	Khorazm	Amu Darya	Unnamed stream	41.540222	60.166666	17/05/2020
22.	Rhodeus_sp_571	SWU09082019571	FFU022-20	MW649174	Acheilognathidae	*Rhodeus* sp.	Sheraliev, BM	Sheraliev, BM	Namangan	Syr Darya	Kara Darya	40.918813	71.828896	09/08/2019
23.	Acipenser_baerii_708	FSU28122019708	FFU023-20	MW649175	Acipenseridae	*Acipenser baerii*	Rozimov, A	Sheraliev, BM	Khorazm	Amu Darya	Amu Darya	41.603361	60.790055	28/12/2019
24.	Acipenser_baerii_709	FSU28122019709	FFU024-20	MW649176	Acipenseridae	*Acipenser baerii*	Rozimov, A	Sheraliev, BM	Khorazm	Amu Darya	Amu Darya	41.603361	60.790055	28/12/2019
25.	Pseudoscaphirhynchus_hermanni_706	FSU19012020706	FFU025-20	MW649177	Acipenseridae	*Pseudoscaphirhynchus hermanni*	Rozimov, A	Sheraliev, BM	Khorazm	Amu Darya	Amu Darya	41.489638	60.955777	19/01/2020
26.	Pseudoscaphirhynchus_hermanni_707	FSU21012020707	FFU026-20	MW649178	Acipenseridae	*Pseudoscaphirhynchus hermanni*	Rozimov, A	Sheraliev, BM	Khorazm	Amu Darya	Amu Darya	41.489638	60.955777	21/01/2020
27.	Pseudoscaphirhynchus_kaufmanni_190	FSU19072018190	FFU027-20	MW649179	Acipenseridae	*Pseudoscaphirhynchus kaufmanni*	Sheraliev, BM	Sheraliev, BM	Khorazm	Amu Darya	Amu Darya	41.466583	60.961305	19/07/2018
28.	Pseudoscaphirhynchus_kaufmanni_678	FSU21082019678	FFU028-20	MW649180	Acipenseridae	*Pseudoscaphirhynchus kaufmanni*	Rozimov, A	Sheraliev, BM	Khorazm	Amu Darya	Amu Darya	41.344972	61.233583	21/08/2019
29.	Pseudoscaphirhynchus_kaufmanni_710	FSU21052020710	FFU029-20	MW649181	Acipenseridae	*Pseudoscaphirhynchus kaufmanni*	Rozimov, A	Sheraliev, BM	Khorazm	Amu Darya	Amu Darya	41.660805	60.689305	21/05/2020
30.	Channa_argus_014	SWU14082016014	FFU030-20	MW649182	Channidae	*Channa argus*	Sheraliev, BM	Sheraliev, BM	Surxondaryo	Amu Darya	South Surkhan R.	37.854013	67.628373	14/08/2016
31.	Channa_argus_092	SWU23012018092	FFU031-20	MW649183	Channidae	*Channa argus*	Sheraliev, BM	Sheraliev, BM	Fergana	Syr Darya	Local fish market			23/01/2018
32.	Channa_argus_093	SWU23012018093	FFU032-20	MW649184	Channidae	*Channa argus*	Sheraliev, BM	Sheraliev, BM	Fergana	Syr Darya	Local fish market			23/01/2018
33.	Channa_argus_094	SWU23012018094	FFU033-20	MW649185	Channidae	*Channa argus*	Sheraliev, BM	Sheraliev, BM	Fergana	Syr Darya	Local fish market			23/01/2018
34.	Channa_argus_095	SWU23012018095	FFU034-20	MW649186	Channidae	*Channa argus*	Sheraliev, BM	Sheraliev, BM	Fergana	Syr Darya	Local fish market			23/01/2018
35.	Channa_argus_096	SWU23012018096	FFU035-20	MW649187	Channidae	*Channa argus*	Sheraliev, BM	Sheraliev, BM	Fergana	Syr Darya	Local fish market			23/01/2018
36.	Sabanejewia_aurata_253	SWU19072019253	FFU036-20	MW649188	Cobitidae	*Sabanejewia aurata*	Sheraliev, BM	Sheraliev, BM	Surxondaryo	Amu Darya	Surkhan Darya	37.255833	67.338611	19/07/2019
37.	Sabanejewia_aurata_254	SWU19072019254	FFU037-20	MW649189	Cobitidae	*Sabanejewia aurata*	Sheraliev, BM	Sheraliev, BM	Surxondaryo	Amu Darya	Surkhan Darya	37.255833	67.338611	19/07/2019
38.	Sabanejewia_aurata_255	SWU19072019255	FFU038-20	MW649190	Cobitidae	*Sabanejewia aurata*	Sheraliev, BM	Sheraliev, BM	Surxondaryo	Amu Darya	Surkhan Darya	37.255833	67.338611	19/07/2019
39.	Sabanejewia_aurata_256	SWU19072019256	FFU039-20	MW649191	Cobitidae	*Sabanejewia aurata*	Sheraliev, BM	Sheraliev, BM	Surxondaryo	Amu Darya	Surkhan Darya	37.255833	67.338611	19/07/2019
40.	Sabanejewia_aurata_300	SWU21072019300	FFU040-20	MW649192	Cobitidae	*Sabanejewia aurata*	Sheraliev, BM	Sheraliev, BM	Surxondaryo	Amu Darya	Surkhan Darya	37.238888	67.329722	21/07/2019
41.	Sabanejewia_aurata_301	SWU21072019301	FFU041-20	MW649193	Cobitidae	*Sabanejewia aurata*	Sheraliev, BM	Sheraliev, BM	Surxondaryo	Amu Darya	Surkhan Darya	37.238888	67.329722	21/07/2019
42.	Sabanejewia_aurata_302	SWU21072019302	FFU042-20	MW649194	Cobitidae	*Sabanejewia aurata*	Sheraliev, BM	Sheraliev, BM	Surxondaryo	Amu Darya	Surkhan Darya	37.238888	67.329722	21/07/2019
43.	Sabanejewia_aurata_308	SWU21072019308	FFU043-20	MW649195	Cobitidae	*Sabanejewia aurata*	Sheraliev, BM	Sheraliev, BM	Surxondaryo	Amu Darya	Surkhan Darya	37.238888	67.329722	21/07/2019
44.	Sabanejewia_aurata_326	SWU22072019326	FFU044-20	MW649196	Cobitidae	*Sabanejewia aurata*	Sheraliev, BM	Sheraliev, BM	Surxondaryo	Amu Darya	Karatag river	38.363055	68.072059	22/07/2019
45.	Sabanejewia_aurata_327	SWU22072019327	FFU045-20	MW649197	Cobitidae	*Sabanejewia aurata*	Sheraliev, BM	Sheraliev, BM	Surxondaryo	Amu Darya	Karatag river	38.363055	68.072059	22/07/2019
46.	Sabanejewia_aurata_328	SWU22072019328	FFU046-20	MW649198	Cobitidae	*Sabanejewia aurata*	Sheraliev, BM	Sheraliev, BM	Surxondaryo	Amu Darya	Karatag river	38.363055	68.072059	22/07/2019
47.	Sabanejewia_aurata_329	SWU22072019329	FFU047-20	MW649199	Cobitidae	*Sabanejewia aurata*	Sheraliev, BM	Sheraliev, BM	Surxondaryo	Amu Darya	Karatag river	38.363055	68.072059	22/07/2019
48.	Sabanejewia_aurata_330	SWU22072019330	FFU048-20	MW649200	Cobitidae	*Sabanejewia aurata*	Sheraliev, BM	Sheraliev, BM	Surxondaryo	Amu Darya	Karatag river	38.363055	68.072059	22/07/2019
49.	Sabanejewia_aurata_384	SWU23072019384	FFU049-20	MW649201	Cobitidae	*Sabanejewia aurata*	Sheraliev, BM	Sheraliev, BM	Surxondaryo	Amu Darya	Tupalang River	38.318966	68.006169	23/07/2019
50.	Sabanejewia_aurata_385	SWU23072019385	FFU050-20	MW649202	Cobitidae	*Sabanejewia aurata*	Sheraliev, BM	Sheraliev, BM	Surxondaryo	Amu Darya	Tupalang River	38.318966	68.006169	23/07/2019
51.	Sabanejewia_aurata_386	SWU23072019386	FFU051-20	MW649203	Cobitidae	*Sabanejewia aurata*	Sheraliev, BM	Sheraliev, BM	Surxondaryo	Amu Darya	Tupalang River	38.318966	68.006169	23/07/2019
52.	Sabanejewia_aurata_387	SWU23072019387	FFU052-20	MW649204	Cobitidae	*Sabanejewia aurata*	Sheraliev, BM	Sheraliev, BM	Surxondaryo	Amu Darya	Tupalang River	38.318966	68.006169	23/07/2019
53.	Sabanejewia_aurata_388	SWU23072019388	FFU053-20	MW649205	Cobitidae	*Sabanejewia aurata*	Sheraliev, BM	Sheraliev, BM	Surxondaryo	Amu Darya	Tupalang River	38.318966	68.006169	23/07/2019
54.	Sabanejewia_aurata_403	SWU25072019403	FFU054-20	MW649206	Cobitidae	*Sabanejewia aurata*	Sheraliev, BM	Sheraliev, BM	Surxondaryo	Amu Darya	Sherobod river	37.747222	66.996666	25/07/2019
55.	Sabanejewia_aurata_416	SWU27072019416	FFU055-20	MW649207	Cobitidae	*Sabanejewia aurata*	Sheraliev, BM	Sheraliev, BM	Bukhara	Zeravshan	Echkiliksoy	40.267551	64.743333	27/07/2019
56.	Sabanejewia_aurata_507	SWU02082019507	FFU056-20	MW649208	Cobitidae	*Sabanejewia aurata*	Sheraliev, BM	Sheraliev, BM	Samarkand	Zeravshan	Zeravshan	39.676388	67.076111	02/08/2019
57.	Sabanejewia_aurata_508	SWU02082019508	FFU057-20	MW649209	Cobitidae	*Sabanejewia aurata*	Sheraliev, BM	Sheraliev, BM	Samarkand	Zeravshan	Zeravshan	39.676388	67.076111	02/08/2019
58.	Sabanejewia_aurata_509	SWU02082019509	FFU058-20	MW649210	Cobitidae	*Sabanejewia aurata*	Sheraliev, BM	Sheraliev, BM	Samarkand	Zeravshan	Zeravshan	39.676388	67.076111	02/08/2019
59.	Sabanejewia_aurata_510	SWU02082019510	FFU059-20	MW649211	Cobitidae	*Sabanejewia aurata*	Sheraliev, BM	Sheraliev, BM	Samarkand	Zeravshan	Zeravshan	39.676388	67.076111	02/08/2019
60.	Sabanejewia_aurata_610	SWU18082019610	FFU060-20	MW649212	Cobitidae	*Sabanejewia aurata*	Sheraliev, BM	Sheraliev, BM	Tashkent	Syr Darya	Chirchik River	41.274444	69.389166	18/08/2019
61.	Sabanejewia_aurata_611	SWU18082019611	FFU061-20	MW649213	Cobitidae	*Sabanejewia aurata*	Sheraliev, BM	Sheraliev, BM	Tashkent	Syr Darya	Chirchik River	41.274444	69.389166	18/08/2019
62.	Sabanejewia_aurata_612	SWU18082019612	FFU062-20	MW649214	Cobitidae	*Sabanejewia aurata*	Sheraliev, BM	Sheraliev, BM	Tashkent	Syr Darya	Chirchik River	41.274444	69.389166	18/08/2019
63.	Cottus_spinulosus_553	SWU09082019553	FFU063-20	MW649215	Cottidae	*Cottus spinulosus*	Sheraliev, BM	Sheraliev, BM	Namangan	Syr Darya	Kara Darya	40.918813	71.828896	09/08/2019
64.	Cottus_spinulosus_554	SWU09082019554	FFU064-20	MW649216	Cottidae	*Cottus spinulosus*	Sheraliev, BM	Sheraliev, BM	Namangan	Syr Darya	Kara Darya	40.918813	71.828896	09/08/2019

65.	Cottus_spinulosus_555	SWU09082019555	FFU065-20	MW649217	Cottidae	*Cottus spinulosus*	Sheraliev, BM	Sheraliev, BM	Namangan	Syr Darya	Kara Darya	40.918813	71.828896	09/08/2019
66.	Cottus_spinulosus_556	SWU09082019556	FFU066-20	MW649218	Cottidae	*Cottus spinulosus*	Sheraliev, BM	Sheraliev, BM	Namangan	Syr Darya	Kara Darya	40.918813	71.828896	09/08/2019
67.	Cottus_spinulosus_557	SWU09082019557	FFU067-20	MW649219	Cottidae	*Cottus spinulosus*	Sheraliev, BM	Sheraliev, BM	Namangan	Syr Darya	Kara Darya	40.918813	71.828896	09/08/2019
68.	Cottus_spinulosus_558	SWU09082019558	FFU068-20	MW649220	Cottidae	*Cottus spinulosus*	Sheraliev, BM	Sheraliev, BM	Namangan	Syr Darya	Kara Darya	40.918813	71.828896	09/08/2019
69.	Capoeta_heratensis_008	SWU10082016008	FFU069-20	MW649221	Cyprinidae	*Capoeta heratensis*	Sheraliev, BM	Sheraliev, BM	Surxondaryo	Amu Darya	Tupalang river	38.399802	67.944766	10/08/2016
70.	Capoeta_heratensis_145	SWU16072018145	FFU070-20	MW649222	Cyprinidae	*Capoeta heratensis*	Sheraliev, BM	Sheraliev, BM	Bukhara	Zeravshan	Zeravshan	40.146944	64.891666	16/07/2018
71.	Capoeta_heratensis_146	SWU16072018146	FFU071-20	MW649223	Cyprinidae	*Capoeta heratensis*	Sheraliev, BM	Sheraliev, BM	Bukhara	Zeravshan	Zeravshan	40.146944	64.891666	16/07/2018
72.	Capoeta_heratensis_147	SWU16072018147	FFU072-20	MW649224	Cyprinidae	*Capoeta heratensis*	Sheraliev, BM	Sheraliev, BM	Bukhara	Zeravshan	Zeravshan	40.146944	64.891666	16/07/2018
73.	Capoeta_heratensis_148	SWU16072018148	FFU073-20	MW649225	Cyprinidae	*Capoeta heratensis*	Sheraliev, BM	Sheraliev, BM	Bukhara	Zeravshan	Zeravshan	40.146944	64.891666	16/07/2018
74.	Capoeta_heratensis_149	SWU16072018149	FFU074-20	MW649226	Cyprinidae	*Capoeta heratensis*	Sheraliev, BM	Sheraliev, BM	Bukhara	Zeravshan	Zeravshan	40.146944	64.891666	16/07/2018
75.	Capoeta_heratensis_169	SWU17072018169	FFU075-20	MW649227	Cyprinidae	*Capoeta heratensis*	Sheraliev, BM	Sheraliev, BM	Bukhara	Zeravshan	Dzhalvan canal	40.149666	64.885400	17/07/2018
76.	Capoeta_heratensis_170	SWU17072018170	FFU076-20	MW649228	Cyprinidae	*Capoeta heratensis*	Sheraliev, BM	Sheraliev, BM	Bukhara	Zeravshan	Dzhalvan canal	40.149666	64.885400	17/07/2018
77.	Capoeta_heratensis_258	SWU19072019258	FFU077-20	MW649229	Cyprinidae	*Capoeta heratensis*	Sheraliev, BM	Sheraliev, BM	Surxondaryo	Amu Darya	Surkhan Darya	37.255833	67.338611	19/07/2019
78.	Capoeta_heratensis_259	SWU19072019259	FFU078-20	MW649230	Cyprinidae	*Capoeta heratensis*	Sheraliev, BM	Sheraliev, BM	Surxondaryo	Amu Darya	Surkhan Darya	37.255833	67.338611	19/07/2019
79.	Capoeta_heratensis_260	SWU19072019260	FFU079-20	MW649231	Cyprinidae	*Capoeta heratensis*	Sheraliev, BM	Sheraliev, BM	Surxondaryo	Amu Darya	Surkhan Darya	37.255833	67.338611	19/07/2019
80.	Capoeta_heratensis_306	SWU21072019307	FFU080-20	MW649232	Cyprinidae	*Capoeta heratensis*	Sheraliev, BM	Sheraliev, BM	Surxondaryo	Amu Darya	Surkhan Darya	37.238888	67.329722	21/07/2019
81.	Capoeta_heratensis_320	SWU21072019320	FFU081-20	MW649233	Cyprinidae	*Capoeta heratensis*	Sheraliev, BM	Sheraliev, BM	Surxondaryo	Amu Darya	Surkhan Darya	37.238888	67.329722	21/07/2019
82.	Capoeta_heratensis_322	SWU21072019322	FFU082-20	MW649234	Cyprinidae	*Capoeta heratensis*	Sheraliev, BM	Sheraliev, BM	Surxondaryo	Amu Darya	Surkhan Darya	37.238888	67.329722	21/07/2019
83.	Capoeta_heratensis_404	SWU25072019404	FFU083-20	MW649235	Cyprinidae	*Capoeta heratensis*	Sheraliev, BM	Sheraliev, BM	Surxondaryo	Amu Darya	Sherobod river	37.747222	66.996666	25/07/2019
84.	Capoeta_heratensis_412	SWU25072019412	FFU084-20	MW649236	Cyprinidae	*Capoeta heratensis*	Sheraliev, BM	Sheraliev, BM	Surxondaryo	Amu Darya	Sherobod river	37.747222	66.996666	25/07/2019
85.	Capoeta_heratensis_413	SWU25072019413	FFU085-20	MW649237	Cyprinidae	*Capoeta heratensis*	Sheraliev, BM	Sheraliev, BM	Surxondaryo	Amu Darya	Sherobod river	37.747222	66.996666	25/07/2019
86.	Capoeta_heratensis_414	SWU25072019414	FFU086-20	MW649238	Cyprinidae	*Capoeta heratensis*	Sheraliev, BM	Sheraliev, BM	Surxondaryo	Amu Darya	Sherobod river	37.747222	66.996666	25/07/2019
87.	Capoeta_heratensis_432	SWU27072019432	FFU087-20	MW649239	Cyprinidae	*Capoeta heratensis*	Sheraliev, BM	Sheraliev, BM	Bukhara	Zeravshan	Echkiliksoy	40.267551	64.743333	27/07/2019
88.	Capoeta_heratensis_433	SWU27072019433	FFU088-20	MW649240	Cyprinidae	*Capoeta heratensis*	Sheraliev, BM	Sheraliev, BM	Bukhara	Zeravshan	Echkiliksoy	40.267551	64.743333	27/07/2019
89.	Capoeta_heratensis_434	SWU27072019434	FFU089-20	MW649241	Cyprinidae	*Capoeta heratensis*	Sheraliev, BM	Sheraliev, BM	Bukhara	Zeravshan	Echkiliksoy	40.267551	64.743333	27/07/2019
90.	Capoeta_heratensis_474	SWU29072019474	FFU090-20	MW649242	Cyprinidae	*Capoeta heratensis*	Sheraliev, BM	Sheraliev, BM	Bukhara	Zeravshan	Unnamed stream	40.147222	64.905805	29/07/2019
91.	Capoeta_heratensis_693	SWU27042019693	FFU091-20	MW649243	Cyprinidae	*Capoeta heratensis*	Allayarov, S	Sheraliev, BM	Surxondaryo	Amu Darya	Karatag river	38.363055	68.072059	27/04/2019
92.	Capoeta_heratensis_694	SWU27042019694	FFU092-20	MW649244	Cyprinidae	*Capoeta heratensis*	Allayarov, S	Sheraliev, BM	Surxondaryo	Amu Darya	Karatag river	38.363055	68.072059	27/04/2019
93.	Capoeta_heratensis_695	SWU27042019695	FFU093-20	MW649245	Cyprinidae	*Capoeta heratensis*	Allayarov, S	Sheraliev, BM	Surxondaryo	Amu Darya	Karatag river	38.363055	68.072059	27/04/2019
94.	Carassius_auratus_134	SWU22022018134	FFU094-20	MW649246	Cyprinidae	*Carassius auratus*	Sheraliev, BM	Sheraliev, BM	Bukhara	Zeravshan	Qoraqir Lake	40.385644	63.308388	22/02/2018
95.	Carassius_auratus_135	SWU22022018135	FFU095-20	MW649247	Cyprinidae	*Carassius auratus*	Sheraliev, BM	Sheraliev, BM	Bukhara	Zeravshan	Qoraqir Lake	40.385644	63.308388	22/02/2018
96.	Carassius_auratus_136	SWU22022018136	FFU096-20	MW649248	Cyprinidae	*Carassius auratus*	Sheraliev, BM	Sheraliev, BM	Bukhara	Zeravshan	Qoraqir Lake	40.385644	63.308388	22/02/2018
97.	Carassius_auratus_160	SWU16072018160	FFU097-20	MW649249	Cyprinidae	*Carassius auratus*	Sheraliev, BM	Sheraliev, BM	Bukhara	Zeravshan	Zeravshan	40.146944	64.891666	16/07/2018
98.	Carassius_auratus_161	SWU16072018161	FFU098-20	MW649250	Cyprinidae	*Carassius auratus*	Sheraliev, BM	Sheraliev, BM	Bukhara	Zeravshan	Zeravshan	40.146944	64.891666	16/07/2018
99.	Carassius_auratus_162	SWU16072018162	FFU099-20	MW649251	Cyprinidae	*Carassius auratus*	Sheraliev, BM	Sheraliev, BM	Bukhara	Zeravshan	Zeravshan	40.146944	64.891666	16/07/2018
100.	Carassius_auratus_164	SWU16072018164	FFU100-20	MW649252	Cyprinidae	*Carassius auratus*	Sheraliev, BM	Sheraliev, BM	Bukhara	Zeravshan	Zeravshan	40.146944	64.891666	16/07/2018
101.	Carassius_gibelio_011	SWU14082016011	FFU101-20	MW649253	Cyprinidae	*Carassius gibelio*	Sheraliev, BM	Sheraliev, BM	Surxondaryo	Amu Darya	South Surkhan R.	37.854013	67.628373	14/08/2016
102.	Carassius_gibelio_012	SWU14082016012	FFU102-20	MW649254	Cyprinidae	*Carassius gibelio*	Sheraliev, BM	Sheraliev, BM	Surxondaryo	Amu Darya	South Surkhan R.	37.854013	67.628373	14/08/2016
103.	Carassius_gibelio_024	SWU16082016024	FFU103-20	MW649255	Cyprinidae	*Carassius gibelio*	Sheraliev, BM	Sheraliev, BM	Qashqadaryo	Amu Darya	Qorasuv Stream	38.570458	66.296166	16/08/2016
104.	Carassius_gibelio_025	SWU16082016025	FFU104-20	MW649256	Cyprinidae	*Carassius gibelio*	Sheraliev, BM	Sheraliev, BM	Qashqadaryo	Amu Darya	Qorasuv Stream	38.570458	66.296166	16/08/2016
105.	Carassius_gibelio_026	SWU16082016026	FFU105-20	MW649257	Cyprinidae	*Carassius gibelio*	Sheraliev, BM	Sheraliev, BM	Qashqadaryo	Amu Darya	Qorasuv Stream	38.570458	66.296166	16/08/2016
106.	Carassius_gibelio_033	SWU31082016033	FFU106-20	MW649258	Cyprinidae	*Carassius gibelio*	Sheraliev, BM	Sheraliev, BM	Namangan	Syr Darya	Syr Darya	40.882777	71.442222	31/08/2016
107.	Carassius_gibelio_034	SWU31082016034	FFU107-20	MW649259	Cyprinidae	*Carassius gibelio*	Sheraliev, BM	Sheraliev, BM	Namangan	Syr Darya	Syr Darya	40.882777	71.442222	31/08/2016
108.	Carassius_gibelio_035	SWU31082016035	FFU108-20	MW649260	Cyprinidae	*Carassius gibelio*	Sheraliev, BM	Sheraliev, BM	Namangan	Syr Darya	Syr Darya	40.882777	71.442222	31/08/2016
109.	Carassius_gibelio_057	SWU07012017057	FFU109-20	MW649261	Cyprinidae	*Carassius gibelio*	Sheraliev, BM	Sheraliev, BM	Fergana	Syr Darya	Unnamed stream	40.366437	71.316907	07/01/2017
110.	Carassius_gibelio_080	SWU10072017080	FFU110-20	MW649262	Cyprinidae	*Carassius gibelio*	Sheraliev, BM	Sheraliev, BM	Fergana	Syr Darya	Unnamed stream	40.588943	71.537333	10/07/2017
111.	Carassius_gibelio_081	SWU10072017081	FFU111-20	MW649263	Cyprinidae	*Carassius gibelio*	Sheraliev, BM	Sheraliev, BM	Fergana	Syr Darya	Unnamed stream	40.588943	71.537333	10/07/2017
112.	Carassius_gibelio_082	SWU10072017082	FFU112-20	MW649264	Cyprinidae	*Carassius gibelio*	Sheraliev, BM	Sheraliev, BM	Fergana	Syr Darya	Unnamed stream	40.588943	71.537333	10/07/2017
113.	Carassius_gibelio_117	SWU01022018117	FFU113-20	MW649265	Cyprinidae	*Carassius gibelio*	Sheraliev, BM	Sheraliev, BM	Fergana	Syr Darya	Unnamed Stream	40.484532	71.434862	01/02/2018
114.	Carassius_gibelio_118	SWU01022018118	FFU114-20	MW649266	Cyprinidae	*Carassius gibelio*	Sheraliev, BM	Sheraliev, BM	Fergana	Syr Darya	Unnamed Stream	40.484532	71.434862	01/02/2018
115.	Carassius_gibelio_119	SWU01022018119	FFU115-20	MW649267	Cyprinidae	*Carassius gibelio*	Sheraliev, BM	Sheraliev, BM	Fergana	Syr Darya	Unnamed Stream	40.484532	71.434862	01/02/2018
116.	Carassius_gibelio_137	SWU22022018137	FFU116-20	MW649268	Cyprinidae	*Carassius gibelio*	Sheraliev, BM	Sheraliev, BM	Bukhara	Zeravshan	Qoraqir Lake	40.385644	63.308388	22/02/2018
117.	Carassius_gibelio_138	SWU22022018138	FFU117-20	MW649269	Cyprinidae	*Carassius gibelio*	Sheraliev, BM	Sheraliev, BM	Bukhara	Zeravshan	Qoraqir Lake	40.385644	63.308388	22/02/2018
118.	Carassius_gibelio_139	SWU22022018139	FFU118-20	MW649270	Cyprinidae	*Carassius gibelio*	Sheraliev, BM	Sheraliev, BM	Bukhara	Zeravshan	Qoraqir Lake	40.385644	63.308388	22/02/2018
119.	Carassius_gibelio_163	SWU16072018163	FFU119-20	MW649271	Cyprinidae	*Carassius gibelio*	Sheraliev, BM	Sheraliev, BM	Bukhara	Zeravshan	Zeravshan	40.146944	64.891666	16/07/2018
120.	Carassius_gibelio_184	SWU18072018184	FFU120-20	MW649272	Cyprinidae	*Carassius gibelio*	Sheraliev, BM	Sheraliev, BM	Navoiy	Zeravshan	Tudakul Lake	39.801388	64.772222	18/07/2018
121.	Carassius_gibelio_223	SWU07012017223	FFU121-20	MW649273	Cyprinidae	*Carassius gibelio*	Sheraliev, BM	Sheraliev, BM	Fergana	Syr Darya	Unnamed stream	40.366437	71.316907	07/01/2017
122.	Carassius_gibelio_224	SWU07012017224	FFU122-20	MW649274	Cyprinidae	*Carassius gibelio*	Sheraliev, BM	Sheraliev, BM	Fergana	Syr Darya	Unnamed stream	40.366437	71.316907	07/01/2017
123.	Carassius_gibelio_225	SWU07012017225	FFU123-20	MW649275	Cyprinidae	*Carassius gibelio*	Sheraliev, BM	Sheraliev, BM	Fergana	Syr Darya	Unnamed stream	40.366437	71.316907	07/01/2017
124.	Carassius_gibelio_583	SWU09082019583	FFU124-20	MW649276	Cyprinidae	*Carassius gibelio*	Sheraliev, BM	Sheraliev, BM	Namangan	Syr Darya	Naryn River	40.940911	71.853631	09/08/2019
125.	Carassius_gibelio_679	SWU27042019679	FFU125-20	MW649277	Cyprinidae	*Carassius gibelio*	Allayarov, S	Sheraliev, BM	Surxondaryo	Amu Darya	Karatag river	38.363055	68.072059	27/04/2019
126.	Carassius_gibelio_680	SWU27042019680	FFU126-20	MW649278	Cyprinidae	*Carassius gibelio*	Allayarov, S	Sheraliev, BM	Surxondaryo	Amu Darya	Karatag river	38.363055	68.072059	27/04/2019
127.	Carassius_gibelio_681	SWU27042019681	FFU127-20	MW649279	Cyprinidae	*Carassius gibelio*	Allayarov, S	Sheraliev, BM	Surxondaryo	Amu Darya	Karatag river	38.363055	68.072059	27/04/2019
128.	Carassius_gibelio_682	SWU27042019681	FFU128-20	MW649280	Cyprinidae	*Carassius gibelio*	Allayarov, S	Sheraliev, BM	Surxondaryo	Amu Darya	Karatag river	38.363055	68.072059	27/04/2019
129.	Cyprinus_carpio_013	SWU14082016013	FFU129-20	MW649281	Cyprinidae	*Cyprinus carpio*	Sheraliev, BM	Sheraliev, BM	Surxondaryo	Amu Darya	South Surkhan R.	37.854013	67.628373	14/08/2016
130.	Cyprinus_carpio_021	SWU16082016021	FFU130-20	MW649282	Cyprinidae	*Cyprinus carpio*	Sheraliev, BM	Sheraliev, BM	Qashqadaryo	Amu Darya	Qorasuv Stream	38.570458	66.296166	16/08/2016
131.	Cyprinus_carpio_022	SWU16082016022	FFU131-20	MW649283	Cyprinidae	*Cyprinus carpio*	Sheraliev, BM	Sheraliev, BM	Qashqadaryo	Amu Darya	Qorasuv Stream	38.570458	66.296166	16/08/2016
132.	Cyprinus_carpio_023	SWU16082016023	FFU132-20	MW649284	Cyprinidae	*Cyprinus carpio*	Sheraliev, BM	Sheraliev, BM	Qashqadaryo	Amu Darya	Qorasuv Stream	38.570458	66.296166	16/08/2016
133.	Cyprinus_carpio_077	SWU10072017077	FFU133-20	MW649285	Cyprinidae	*Cyprinus carpio*	Sheraliev, BM	Sheraliev, BM	Fergana	Syr Darya	Unnamed stream	40.588943	71.537333	10/07/2017
134.	Cyprinus_carpio_078	SWU10072017078	FFU134-20	MW649286	Cyprinidae	*Cyprinus carpio*	Sheraliev, BM	Sheraliev, BM	Fergana	Syr Darya	Unnamed stream	40.588943	71.537333	10/07/2017
135.	Cyprinus_carpio_079	SWU10072017079	FFU135-20	MW649287	Cyprinidae	*Cyprinus carpio*	Sheraliev, BM	Sheraliev, BM	Fergana	Syr Darya	Unnamed stream	40.588943	71.537333	10/07/2017
136.	Cyprinus_carpio_154	SWU16072018154	FFU136-20	MW649288	Cyprinidae	*Cyprinus carpio*	Sheraliev, BM	Sheraliev, BM	Bukhara	Zeravshan	Zeravshan	40.146944	64.891666	16/07/2018
137.	Cyprinus_carpio_183	SWU18072018183	FFU137-20	MW649289	Cyprinidae	*Cyprinus carpio*	Sheraliev, BM	Sheraliev, BM	Navoiy	Zeravshan	Tudakul Lake	39.801388	64.772222	18/07/2018
138.	Luciobarbus_brachycephalus_483	SWU31072019483	FFU138-20	MW649290	Cyprinidae	*Luciobarbus brachycephalus*	Sheraliev, BM	Sheraliev, BM	Khorazm	Amu Darya	Amu Darya	41.650833	60.704722	31/07/2019
139.	Luciobarbus_brachycephalus_484	SWU31072019484	FFU139-20	MW649291	Cyprinidae	*Luciobarbus brachycephalus*	Sheraliev, BM	Sheraliev, BM	Khorazm	Amu Darya	Amu Darya	41.650833	60.704722	31/07/2019
140.	Luciobarbus_brachycephalus_485	SWU31072019485	FFU140-20	MW649292	Cyprinidae	*Luciobarbus brachycephalus*	Sheraliev, BM	Sheraliev, BM	Khorazm	Amu Darya	Amu Darya	41.650833	60.704722	31/07/2019
141.	Luciobarbus_brachycephalus_486	SWU31072019486	FFU141-20	MW649293	Cyprinidae	*Luciobarbus brachycephalus*	Sheraliev, BM	Sheraliev, BM	Khorazm	Amu Darya	Amu Darya	41.650833	60.704722	31/07/2019

142.	Luciobarbus_brachycephalus_487	SWU31072019487	FFU142-20	MW649294	Cyprinidae	*Luciobarbus brachycephalus*	Sheraliev, BM	Sheraliev, BM	Khorazm	Amu Darya	Amu Darya	41.650833	60.704722	31/07/2019
143.	Luciobarbus_brachycephalus_488	SWU31072019488	FFU143-20	MW649295	Cyprinidae	*Luciobarbus brachycephalus*	Sheraliev, BM	Sheraliev, BM	Khorazm	Amu Darya	Amu Darya	41.650833	60.704722	31/07/2019
144.	Luciobarbus_brachycephalus_670	SWU21082019670	FFU144-20	MW649296	Cyprinidae	*Luciobarbus brachycephalus*	Rozimov, A	Sheraliev, BM	Khorazm	Amu Darya	Amu Darya	41.650833	60.704722	21/08/2019
145.	Luciobarbus_brachycephalus_671	SWU21082019671	FFU145-20	MW649297	Cyprinidae	*Luciobarbus brachycephalus*	Rozimov, A	Sheraliev, BM	Khorazm	Amu Darya	Amu Darya	41.650833	60.704722	21/08/2019
146.	Luciobarbus_conocephalus_041	SWU31082016041	FFU146-20	MW649298	Cyprinidae	*Luciobarbus conocephalus*	Sheraliev, BM	Sheraliev, BM	Namangan	Syr Darya	Syr Darya	40.882777	71.442222	31/08/2016
147.	Luciobarbus_conocephalus_042	SWU31082016042	FFU147-20	MW649299	Cyprinidae	*Luciobarbus conocephalus*	Sheraliev, BM	Sheraliev, BM	Namangan	Syr Darya	Syr Darya	40.882777	71.442222	31/08/2016
148.	Luciobarbus_conocephalus_044	SWU31082016044	FFU148-21	MW649300	Cyprinidae	*Luciobarbus conocephalus*	Sheraliev, BM	Sheraliev, BM	Namangan	Syr Darya	Syr Darya	40.882777	71.442222	31/08/2016
149.	Luciobarbus_conocephalus_045	SWU31082016045	FFU149-21	MW649301	Cyprinidae	*Luciobarbus conocephalus*	Sheraliev, BM	Sheraliev, BM	Namangan	Syr Darya	Syr Darya	40.882777	71.442222	31/08/2016
150.	Luciobarbus_conocephalus_046	SWU31082016046	FFU150-21	MW649302	Cyprinidae	*Luciobarbus conocephalus*	Sheraliev, BM	Sheraliev, BM	Namangan	Syr Darya	Syr Darya	40.882777	71.442222	31/08/2016
151.	Luciobarbus_conocephalus_125	SWU08022018125	FFU151-21	MW649303	Cyprinidae	*Luciobarbus conocephalus*	Sheraliev, BM	Sheraliev, BM	Fergana	Syr Darya	Sokh River	40.340176	71.013559	08/02/2018
152.	Luciobarbus_conocephalus_171	SWU17072018171	FFU152-21	MW649304	Cyprinidae	*Luciobarbus conocephalus*	Sheraliev, BM	Sheraliev, BM	Bukhara	Zeravshan	Dzhalvan canal	40.149666	64.885400	17/07/2018
153.	Luciobarbus_conocephalus_172	SWU17072018172	FFU153-21	MW649305	Cyprinidae	*Luciobarbus conocephalus*	Sheraliev, BM	Sheraliev, BM	Bukhara	Zeravshan	Dzhalvan canal	40.149666	64.885400	17/07/2018
154.	Luciobarbus_conocephalus_229	SWU31082016229	FFU154-21	MW649306	Cyprinidae	*Luciobarbus conocephalus*	Sheraliev, BM	Sheraliev, BM	Namangan	Syr Darya	Syr Darya	40.882777	71.442222	31/08/2016
155.	Luciobarbus_conocephalus_310	SWU21072019310	FFU155-21	MW649307	Cyprinidae	*Luciobarbus conocephalus*	Sheraliev, BM	Sheraliev, BM	Surxondaryo	Amu Darya	Surkhan Darya	37.238888	67.329722	21/07/2019
156.	Luciobarbus_conocephalus_311	SWU21072019311	FFU156-21	MW649308	Cyprinidae	*Luciobarbus conocephalus*	Sheraliev, BM	Sheraliev, BM	Surxondaryo	Amu Darya	Surkhan Darya	37.238888	67.329722	21/07/2019
157.	Luciobarbus_conocephalus_312	SWU21072019312	FFU157-21	MW649309	Cyprinidae	*Luciobarbus conocephalus*	Sheraliev, BM	Sheraliev, BM	Surxondaryo	Amu Darya	Surkhan Darya	37.238888	67.329722	21/07/2019
158.	Luciobarbus_conocephalus_331	SWU22072019331	FFU158-21	MW649310	Cyprinidae	*Luciobarbus conocephalus*	Sheraliev, BM	Sheraliev, BM	Surxondaryo	Amu Darya	Karatag river	38.363055	68.072059	22/07/2019
159.	Luciobarbus_conocephalus_332	SWU22072019332	FFU159-21	MW649311	Cyprinidae	*Luciobarbus conocephalus*	Sheraliev, BM	Sheraliev, BM	Surxondaryo	Amu Darya	Karatag river	38.363055	68.072059	22/07/2019
160.	Luciobarbus_conocephalus_333	SWU22072019333	FFU160-21	MW649312	Cyprinidae	*Luciobarbus conocephalus*	Sheraliev, BM	Sheraliev, BM	Surxondaryo	Amu Darya	Karatag river	38.363055	68.072059	22/07/2019
161.	Schizothorax_eurystomus_043	SWU31082016043	FFU161-21	MW649313	Cyprinidae	*Schizothorax eurystomus*	Sheraliev, BM	Sheraliev, BM	Namangan	Syr Darya	Syr Darya	40.882777	71.442222	31/08/2016
162.	Schizothorax_eurystomus_047	SWU31082016047	FFU162-21	MW649314	Cyprinidae	*Schizothorax eurystomus*	Sheraliev, BM	Sheraliev, BM	Namangan	Syr Darya	Syr Darya	40.882777	71.442222	31/08/2016
163.	Schizothorax_eurystomus_048	SWU31082016048	FFU163-21	MW649315	Cyprinidae	*Schizothorax eurystomus*	Sheraliev, BM	Sheraliev, BM	Namangan	Syr Darya	Syr Darya	40.882777	71.442222	31/08/2016
164.	Schizothorax_eurystomus_049	SWU31082016049	FFU164-21	MW649316	Cyprinidae	*Schizothorax eurystomus*	Sheraliev, BM	Sheraliev, BM	Namangan	Syr Darya	Syr Darya	40.882777	71.442222	31/08/2016
165.	Schizothorax_eurystomus_058	SWU07012017058	FFU165-21	MW649317	Cyprinidae	*Schizothorax eurystomus*	Sheraliev, BM	Sheraliev, BM	Fergana	Syr Darya	Unnamed stream	40.366437	71.316907	07/01/2017
166.	Schizothorax_eurystomus_120	SWU08022018120	FFU166-21	MW649318	Cyprinidae	*Schizothorax eurystomus*	Sheraliev, BM	Sheraliev, BM	Fergana	Syr Darya	Sokh River	40.340176	71.013559	08/02/2018
167.	Schizothorax_eurystomus_121	SWU08022018121	FFU167-21	MW649319	Cyprinidae	*Schizothorax eurystomus*	Sheraliev, BM	Sheraliev, BM	Fergana	Syr Darya	Sokh River	40.340176	71.013559	08/02/2018
168.	Schizothorax_eurystomus_122	SWU08022018122	FFU168-21	MW649320	Cyprinidae	*Schizothorax eurystomus*	Sheraliev, BM	Sheraliev, BM	Fergana	Syr Darya	Sokh River	40.340176	71.013559	08/02/2018
169.	Schizothorax_eurystomus_123	SWU08022018123	FFU169-21	MW649321	Cyprinidae	*Schizothorax eurystomus*	Sheraliev, BM	Sheraliev, BM	Fergana	Syr Darya	Sokh River	40.340176	71.013559	08/02/2018
170.	Schizothorax_eurystomus_124	SWU08022018124	FFU170-21	MW649322	Cyprinidae	*Schizothorax eurystomus*	Sheraliev, BM	Sheraliev, BM	Fergana	Syr Darya	Sokh River	40.340176	71.013559	08/02/2018
171.	Schizothorax_eurystomus_198	SWU11082018198	FFU171-21	MW649323	Cyprinidae	*Schizothorax eurystomus*	Sheraliev, BM	Sheraliev, BM	Fergana	Syr Darya	Unnamed stream	40.312769	71.811647	11/08/2018
172.	Schizothorax_eurystomus_199	SWU11082018199	FFU172-21	MW649324	Cyprinidae	*Schizothorax eurystomus*	Sheraliev, BM	Sheraliev, BM	Fergana	Syr Darya	Unnamed stream	40.312769	71.811647	11/08/2018
173.	Schizothorax_eurystomus_200	SWU11082018200	FFU173-21	MW649325	Cyprinidae	*Schizothorax eurystomus*	Sheraliev, BM	Sheraliev, BM	Fergana	Syr Darya	Unnamed stream	40.312769	71.811647	11/08/2018
174.	Schizothorax_eurystomus_201	SWU11082018201	FFU174-21	MW649326	Cyprinidae	*Schizothorax eurystomus*	Sheraliev, BM	Sheraliev, BM	Fergana	Syr Darya	Unnamed stream	40.312769	71.811647	11/08/2018
175.	Schizothorax_eurystomus_202	SWU11082018202	FFU175-21	MW649327	Cyprinidae	*Schizothorax eurystomus*	Sheraliev, BM	Sheraliev, BM	Fergana	Syr Darya	Unnamed stream	40.312769	71.811647	11/08/2018
176.	Schizothorax_eurystomus_203	SWU11082018203	FFU176-21	MW649328	Cyprinidae	*Schizothorax eurystomus*	Sheraliev, BM	Sheraliev, BM	Fergana	Syr Darya	Unnamed stream	40.312769	71.811647	11/08/2018
177.	Schizothorax_eurystomus_211	SWU15082018211	FFU177-21	MW649329	Cyprinidae	*Schizothorax eurystomus*	Sheraliev, BM	Sheraliev, BM	Fergana	Syr Darya	Shohimardon River	39.963088	71.759857	15/08/2018
178.	Schizothorax_eurystomus_212	SWU15082018212	FFU178-21	MW649330	Cyprinidae	*Schizothorax eurystomus*	Sheraliev, BM	Sheraliev, BM	Fergana	Syr Darya	Shohimardon River	39.963088	71.759857	15/08/2018
179.	Schizothorax_eurystomus_213	SWU15082018213	FFU179-21	MW649331	Cyprinidae	*Schizothorax eurystomus*	Sheraliev, BM	Sheraliev, BM	Fergana	Syr Darya	Shohimardon River	39.963088	71.759857	15/08/2018
180.	Schizothorax_eurystomus_214	SWU15082018214	FFU180-21	MW649332	Cyprinidae	*Schizothorax eurystomus*	Sheraliev, BM	Sheraliev, BM	Fergana	Syr Darya	Shohimardon River	39.963088	71.759857	15/08/2018
181.	Schizothorax_eurystomus_215	SWU15082018215	FFU181-21	MW649333	Cyprinidae	*Schizothorax eurystomus*	Sheraliev, BM	Sheraliev, BM	Fergana	Syr Darya	Shohimardon River	39.963088	71.759857	15/08/2018
182.	Schizothorax_eurystomus_226	SWU15012017226	FFU182-21	MW649334	Cyprinidae	*Schizothorax eurystomus*	Sheraliev, BM	Sheraliev, BM	Fergana	Syr Darya	Unnamed stream	40.326408	71.820508	15/01/2017
183.	Schizothorax_eurystomus_227	SWU15012017227	FFU183-21	MW649335	Cyprinidae	*Schizothorax eurystomus*	Sheraliev, BM	Sheraliev, BM	Fergana	Syr Darya	Unnamed stream	40.326408	71.820508	15/01/2017
184.	Schizothorax_eurystomus_228	SWU15012017228	FFU184-21	MW649336	Cyprinidae	*Schizothorax eurystomus*	Sheraliev, BM	Sheraliev, BM	Fergana	Syr Darya	Unnamed stream	40.326408	71.820508	15/01/2017
185.	Schizothorax_eurystomus_542	SWU05082019542	FFU185-21	MW649337	Cyprinidae	*Schizothorax eurystomus*	Bakhromova, B	Sheraliev, BM	Fergana	Syr Darya	Sokh River	39.980157	71.118103	05/08/2019
186.	Schizothorax_eurystomus_563	SWU09082019563	FFU186-21	MW649338	Cyprinidae	*Schizothorax eurystomus*	Sheraliev, BM	Sheraliev, BM	Namangan	Syr Darya	Kara Darya	40.918813	71.828896	09/08/2019
187.	Schizothorax_eurystomus_564	SWU09082019564	FFU187-21	MW649339	Cyprinidae	*Schizothorax eurystomus*	Sheraliev, BM	Sheraliev, BM	Namangan	Syr Darya	Kara Darya	40.918813	71.828896	09/08/2019
188.	Schizothorax_eurystomus_565	SWU09082019565	FFU188-21	MW649340	Cyprinidae	*Schizothorax eurystomus*	Sheraliev, BM	Sheraliev, BM	Namangan	Syr Darya	Kara Darya	40.918813	71.828896	09/08/2019
189.	Schizothorax_eurystomus_566	SWU09082019566	FFU189-21	MW649341	Cyprinidae	*Schizothorax eurystomus*	Sheraliev, BM	Sheraliev, BM	Namangan	Syr Darya	Kara Darya	40.918813	71.828896	09/08/2019
190.	Schizothorax_eurystomus_567	SWU09082019567	FFU190-21	MW649342	Cyprinidae	*Schizothorax eurystomus*	Sheraliev, BM	Sheraliev, BM	Namangan	Syr Darya	Kara Darya	40.918813	71.828896	09/08/2019
191.	Schizothorax_eurystomus_577	SWU09082019577	FFU191-21	MW649343	Cyprinidae	*Schizothorax eurystomus*	Sheraliev, BM	Sheraliev, BM	Namangan	Syr Darya	Kara Darya	40.918813	71.828896	09/08/2019
192.	Schizothorax_eurystomus_578	SWU09082019578	FFU192-21	MW649344	Cyprinidae	*Schizothorax eurystomus*	Sheraliev, BM	Sheraliev, BM	Namangan	Syr Darya	Kara Darya	40.918813	71.828896	09/08/2019
193.	Schizothorax_eurystomus_584	SWU09082019584	FFU193-21	MW649345	Cyprinidae	*Schizothorax eurystomus*	Sheraliev, BM	Sheraliev, BM	Namangan	Syr Darya	Naryn River	40.940911	71.853631	09/08/2019
194.	Schizothorax_eurystomus_595	SWU18082019595	FFU194-21	MW649346	Cyprinidae	*Schizothorax eurystomus*	Sheraliev, BM	Sheraliev, BM	Tashkent	Syr Darya	Chirchik river	41.274444	69.389166	18/08/2019
195.	Schizothorax_eurystomus_596	SWU18082019596	FFU195-21	MW649347	Cyprinidae	*Schizothorax eurystomus*	Sheraliev, BM	Sheraliev, BM	Tashkent	Syr Darya	Chirchik river	41.274444	69.389166	18/08/2019
196.	Schizothorax_eurystomus_597	SWU18082019597	FFU196-21	MW649348	Cyprinidae	*Schizothorax eurystomus*	Sheraliev, BM	Sheraliev, BM	Tashkent	Syr Darya	Chirchik river	41.274444	69.389166	18/08/2019
197.	Schizothorax_eurystomus_644	SWU20082019644	FFU197-21	MW649349	Cyprinidae	*Schizothorax eurystomus*	Sheraliev, BM	Sheraliev, BM	Fergana	Syr Darya	Unnamed stream	40.305260	71.800688	20/08/2019
198.	Schizothorax_eurystomus_645	SWU20082019645	FFU198-21	MW649350	Cyprinidae	*Schizothorax eurystomus*	Sheraliev, BM	Sheraliev, BM	Fergana	Syr Darya	Unnamed stream	40.305260	71.800688	20/08/2019
199.	Schizothorax_fedtschenkoi_504	SWU02082019504	FFU199-21	MW649351	Cyprinidae	*Schizothorax fedtschenkoi*	Sheraliev, BM	Sheraliev, BM	Samarkand	Zeravshan	Zeravshan	39.676388	67.076111	02/08/2019
200.	Schizothorax_fedtschenkoi_505	SWU02082019505	FFU200-21	MW649352	Cyprinidae	*Schizothorax fedtschenkoi*	Sheraliev, BM	Sheraliev, BM	Samarkand	Zeravshan	Zeravshan	39.676388	67.076111	02/08/2019
201.	Schizothorax_fedtschenkoi_506	SWU02082019506	FFU201-21	MW649353	Cyprinidae	*Schizothorax fedtschenkoi*	Sheraliev, BM	Sheraliev, BM	Samarkand	Zeravshan	Zeravshan	39.676388	67.076111	02/08/2019
202.	Schizothorax_fedtschenkoi_511	SWU02082019511	FFU202-21	MW649354	Cyprinidae	*Schizothorax fedtschenkoi*	Sheraliev, BM	Sheraliev, BM	Samarkand	Zeravshan	Zeravshan	39.676388	67.076111	02/08/2019
203.	Schizothorax_fedtschenkoi_512	SWU02082019512	FFU203-21	MW649355	Cyprinidae	*Schizothorax fedtschenkoi*	Sheraliev, BM	Sheraliev, BM	Samarkand	Zeravshan	Zeravshan	39.676388	67.076111	02/08/2019
204.	Schizothorax_sp_006	SWU08082016006	FFU662-21	MW649356	Cyprinidae	*Schizothorax* sp.	Sheraliev, BM	Sheraliev, BM	Surxondaryo	Amu Darya	Tupalang river	38.399802	67.944766	08/08/2016
205.	Schizothorax_sp_007	SWU08082016007	FFU663-21	MW649357	Cyprinidae	*Schizothorax* sp.	Sheraliev, BM	Sheraliev, BM	Surxondaryo	Amu Darya	Tupalang river	38.399802	67.944766	08/08/2016
206.	Schizothorax_sp_235	SWU12082016235	FFU664-21	MW649358	Cyprinidae	*Schizothorax* sp.	Sheraliev, BM	Sheraliev, BM	Surxondaryo	Amu Darya	Tupalang river	38.399802	67.944766	12/08/2016
207.	Schizothorax_sp_402	SWU25072019402	FFU665-21	MW649359	Cyprinidae	*Schizothorax* sp.	Sheraliev, BM	Sheraliev, BM	Surxondaryo	Amu Darya	Sherobod river	37.747222	66.996666	25/07/2019
208.	Schizothorax_sp_415	SWU24072019415	FFU666-21	MW649360	Cyprinidae	*Schizothorax* sp.	Sheraliev, BM	Sheraliev, BM	Surxondaryo	Amu Darya	Sayrob spring	38.073178	66.968351	24/07/2019
209.	Esox_lucius_701	SWU09112019701	FFU204-21	MW649361	Esocidae	*Esox lucius*	Rozimov, A	Sheraliev, BM	Khorazm	Amu Darya	Unnamed stream	41.488388	60.828055	09/11/2019
210.	Esox_lucius_702	SWU13112010702	FFU205-21	MW649362	Esocidae	*Esox lucius*	Rozimov, A	Sheraliev, BM	Khorazm	Amu Darya	Unnamed stream	41.488388	60.828055	13/11/2019
211.	Esox_lucius_703	SWU13112019703	FFU206-21	MW649363	Esocidae	*Esox lucius*	Rozimov, A	Sheraliev, BM	Khorazm	Amu Darya	Unnamed stream	41.488388	60.828055	13/11/2019
212.	Neogobius_melanostomus_716	SWU18052020716	FFU207-21	MW649364	Gobiidae	*Neogobius melanostonus*	Rozimov, A	Sheraliev, BM	Karakalpakstan	Amu Darya	Central canal	42.635638	59.806944	18/05/2020
213.	Neogobius_pallasi_717	SWU18052020717	FFU208-21	MW649365	Gobiidae	*Neogobius pallasi*	Rozimov, A	Sheraliev, BM	Karakalpakstan	Amu Darya	Central canal	42.635638	59.806944	18/05/2020
214.	Rhinogobius_sp_264	SWU19072019264	FFU209-21	MW649366	Gobiidae	*Rhinogobius* sp.	Sheraliev, BM	Sheraliev, BM	Surxondaryo	Amu Darya	Surkhan Darya	37.255833	67.338611	19/07/2019
215.	Rhinogobius_sp_265	SWU19072019265	FFU210-21	MW649367	Gobiidae	*Rhinogobius* sp.	Sheraliev, BM	Sheraliev, BM	Surxondaryo	Amu Darya	Surkhan Darya	37.255833	67.338611	19/07/2019
216.	Rhinogobius_sp_339	SWU22072019339	FFU211-21	MW649368	Gobiidae	*Rhinogobius* sp.	Sheraliev, BM	Sheraliev, BM	Surxondaryo	Amu Darya	Karatag river	38.363055	68.072059	22/07/2019
217.	Rhinogobius_sp_340	SWU22072019340	FFU212-21	MW649369	Gobiidae	*Rhinogobius* sp.	Sheraliev, BM	Sheraliev, BM	Surxondaryo	Amu Darya	Karatag river	38.363055	68.072059	22/07/2019
218.	Rhinogobius_sp_341	SWU22072019341	FFU213-21	MW649370	Gobiidae	*Rhinogobius* sp.	Sheraliev, BM	Sheraliev, BM	Surxondaryo	Amu Darya	Karatag river	38.363055	68.072059	22/07/2019

219.	Rhinogobius_sp_342	SWU22072019342	FFU214-21	MW649371	Gobiidae	*Rhinogobius* sp.	Sheraliev, BM	Sheraliev, BM	Surxondaryo	Amu Darya	Karatag river	38.363055	68.072059	22/07/2019
220.	Rhinogobius_sp_343	SWU22072019343	FFU215-21	MW649372	Gobiidae	*Rhinogobius* sp.	Sheraliev, BM	Sheraliev, BM	Surxondaryo	Amu Darya	Karatag river	38.363055	68.072059	22/07/2019
221.	Rhinogobius_sp_397	SWU24072019397	FFU216-21	MW649373	Gobiidae	*Rhinogobius* sp.	Sheraliev, BM	Sheraliev, BM	Surxondaryo	Amu Darya	Surkhan Darya	37.823333	67.618611	24/07/2019
222.	Rhinogobius_sp_398	SWU24072019398	FFU217-21	MW649374	Gobiidae	*Rhinogobius* sp.	Sheraliev, BM	Sheraliev, BM	Surxondaryo	Amu Darya	Surkhan Darya	37.823333	67.618611	24/07/2019
223.	Rhinogobius_sp_399	SWU24072019399	FFU218-21	MW649375	Gobiidae	*Rhinogobius* sp.	Sheraliev, BM	Sheraliev, BM	Surxondaryo	Amu Darya	Surkhan Darya	37.823333	67.618611	24/07/2019
224.	Rhinogobius_sp_400	SWU24072019400	FFU219-21	MW649376	Gobiidae	*Rhinogobius* sp.	Sheraliev, BM	Sheraliev, BM	Surxondaryo	Amu Darya	Surkhan Darya	37.823333	67.618611	24/07/2019
225.	Rhinogobius_sp_417	SWU27072019417	FFU220-21	MW649377	Gobiidae	*Rhinogobius* sp.	Sheraliev, BM	Sheraliev, BM	Bukhara	Zeravshan	Echkiliksoy	40.267551	64.743333	27/07/2019
226.	Rhinogobius_sp_418	SWU27072019418	FFU221-21	MW649378	Gobiidae	*Rhinogobius* sp.	Sheraliev, BM	Sheraliev, BM	Bukhara	Zeravshan	Echkiliksoy	40.267551	64.743333	27/07/2019
227.	Rhinogobius_sp_419	SWU27072019419	FFU222-21	MW649379	Gobiidae	*Rhinogobius* sp.	Sheraliev, BM	Sheraliev, BM	Bukhara	Zeravshan	Echkiliksoy	40.267551	64.743333	27/07/2019
228.	Rhinogobius_sp_420	SWU27072019420	FFU223-21	MW649380	Gobiidae	*Rhinogobius* sp.	Sheraliev, BM	Sheraliev, BM	Bukhara	Zeravshan	Echkiliksoy	40.267551	64.743333	27/07/2019
229.	Rhinogobius_sp_437	SWU27072019437	FFU224-21	MW649381	Gobiidae	*Rhinogobius* sp.	Sheraliev, BM	Sheraliev, BM	Bukhara	Zeravshan	Echkiliksoy	40.267551	64.743333	27/07/2019
230.	Rhinogobius_sp_438	SWU27072019438	FFU225-21	MW649382	Gobiidae	*Rhinogobius* sp.	Sheraliev, BM	Sheraliev, BM	Bukhara	Zeravshan	Echkiliksoy	40.267551	64.743333	27/07/2019
231.	Rhinogobius_sp_459	SWU28072019459	FFU226-21	MW649383	Gobiidae	*Rhinogobius* sp.	Sheraliev, BM	Sheraliev, BM	Bukhara	Zeravshan	Zeravshan	40.071012	64.778429	28/07/2019
232.	Rhinogobius_sp_467	SWU28072019467	FFU227-21	MW649384	Gobiidae	*Rhinogobius* sp.	Sheraliev, BM	Sheraliev, BM	Bukhara	Zeravshan	Zeravshan	40.071012	64.778429	28/07/2019
233.	Rhinogobius_sp_468	SWU28072019468	FFU228-21	MW649385	Gobiidae	*Rhinogobius* sp.	Sheraliev, BM	Sheraliev, BM	Bukhara	Zeravshan	Zeravshan	40.071012	64.778429	28/07/2019
234.	Rhinogobius_sp_493	SWU31072019493	FFU229-21	MW649386	Gobiidae	*Rhinogobius* sp.	Sheraliev, BM	Sheraliev, BM	Khorazm	Amu Darya	Amu Darya	41.650833	60.704722	31/07/2019
235.	Rhinogobius_sp_494	SWU31072019494	FFU230-21	MW649387	Gobiidae	*Rhinogobius* sp.	Sheraliev, BM	Sheraliev, BM	Khorazm	Amu Darya	Amu Darya	41.650833	60.704722	31/07/2019
236.	Rhinogobius_sp_495	SWU31072019495	FFU231-21	MW649388	Gobiidae	*Rhinogobius* sp.	Sheraliev, BM	Sheraliev, BM	Khorazm	Amu Darya	Amu Darya	41.650833	60.704722	31/07/2019
237.	Rhinogobius_sp_496	SWU31072019496	FFU232-21	MW649389	Gobiidae	*Rhinogobius* sp.	Sheraliev, BM	Sheraliev, BM	Khorazm	Amu Darya	Amu Darya	41.650833	60.704722	31/07/2019
238.	Rhinogobius_sp_497	SWU31072019497	FFU233-21	MW649390	Gobiidae	*Rhinogobius* sp.	Sheraliev, BM	Sheraliev, BM	Khorazm	Amu Darya	Amu Darya	41.650833	60.704722	31/07/2019
239.	Rhinogobius_sp_543	SWU09082019543	FFU234-21	MW649391	Gobiidae	*Rhinogobius* sp.	Sheraliev, BM	Sheraliev, BM	Namangan	Syr Darya	Kara Darya	40.918813	71.828896	09/08/2019
240.	Rhinogobius_sp_544	SWU09082019544	FFU235-21	MW649392	Gobiidae	*Rhinogobius* sp.	Sheraliev, BM	Sheraliev, BM	Namangan	Syr Darya	Kara Darya	40.918813	71.828896	09/08/2019
241.	Rhinogobius_sp_545	SWU09082019545	FFU236-21	MW649393	Gobiidae	*Rhinogobius* sp.	Sheraliev, BM	Sheraliev, BM	Namangan	Syr Darya	Kara Darya	40.918813	71.828896	09/08/2019
242.	Rhinogobius_sp_546	SWU09082019546	FFU237-21	MW649394	Gobiidae	*Rhinogobius* sp.	Sheraliev, BM	Sheraliev, BM	Namangan	Syr Darya	Kara Darya	40.918813	71.828896	09/08/2019
243.	Rhinogobius_sp_547	SWU09082019547	FFU238-21	MW649395	Gobiidae	*Rhinogobius* sp.	Sheraliev, BM	Sheraliev, BM	Namangan	Syr Darya	Kara Darya	40.918813	71.828896	09/08/2019
244.	Rhinogobius_sp_549	SWU09082019549	FFU239-21	MW649396	Gobiidae	*Rhinogobius* sp.	Sheraliev, BM	Sheraliev, BM	Namangan	Syr Darya	Kara Darya	40.918813	71.828896	09/08/2019
245.	Rhinogobius_sp_586	SWU10082019586	FFU240-21	MW649397	Gobiidae	*Rhinogobius* sp.	Sheraliev, BM	Sheraliev, BM	Namangan	Syr Darya	Syr Darya	40.881008	71.686687	10/08/2019
246.	Rhinogobius_sp_587	SWU10082019587	FFU241-21	MW649398	Gobiidae	*Rhinogobius* sp.	Sheraliev, BM	Sheraliev, BM	Namangan	Syr Darya	Syr Darya	40.881008	71.686687	10/08/2019
247.	Rhinogobius_sp_588	SWU10082019588	FFU242-21	MW649399	Gobiidae	*Rhinogobius* sp.	Sheraliev, BM	Sheraliev, BM	Namangan	Syr Darya	Syr Darya	40.881008	71.686687	10/08/2019
248.	Rhinogobius_sp_589	SWU10082019589	FFU243-21	MW649400	Gobiidae	*Rhinogobius* sp.	Sheraliev, BM	Sheraliev, BM	Namangan	Syr Darya	Syr Darya	40.881008	71.686687	10/08/2019
249.	Rhinogobius_sp_590	SWU10082019590	FFU244-21	MW649401	Gobiidae	*Rhinogobius* sp.	Sheraliev, BM	Sheraliev, BM	Namangan	Syr Darya	Syr Darya	40.881008	71.686687	10/08/2019
250.	Rhinogobius_sp_613	SWU18082019613	FFU245-21	MW649402	Gobiidae	*Rhinogobius* sp.	Sheraliev, BM	Sheraliev, BM	Tashkent	Syr Darya	Chirchik River	41.274444	69.389166	18/08/2019
251.	Abbottina_rivularis_059	SWU07012017059	FFU246-21	MW649403	Gobionidae	*Abbottina rivularis*	Sheraliev, BM	Sheraliev, BM	Fergana	Syr Darya	Unnamed stream	40.366437	71.316907	07/01/2017
252.	Abbottina_rivularis_060	SWU07012017060	FFU247-21	MW649404	Gobionidae	*Abbottina rivularis*	Sheraliev, BM	Sheraliev, BM	Fergana	Syr Darya	Unnamed stream	40.366437	71.316907	07/01/2017
253.	Abbottina_rivularis_153	SWU16072018153	FFU248-21	MW649405	Gobionidae	*Abbottina rivularis*	Sheraliev, BM	Sheraliev, BM	Bukhara	Zeravshan	Zeravshan	40.146944	64.891666	16/07/2018
254.	Abbottina_rivularis_285	SWU20072019285	FFU249-21	MW649406	Gobionidae	*Abbottina rivularis*	Sheraliev, BM	Sheraliev, BM	Surxondaryo	Amu Darya	Amu Darya	37.235555	67.660555	20/07/2019
255.	Abbottina_rivularis_338	SWU22072019338	FFU250-21	MW649407	Gobionidae	*Abbottina rivularis*	Sheraliev, BM	Sheraliev, BM	Surxondaryo	Amu Darya	Karatag river	38.363055	68.072059	22/07/2019
256.	Abbottina_rivularis_428	SWU27072019428	FFU251-21	MW649408	Gobionidae	*Abbottina rivularis*	Sheraliev, BM	Sheraliev, BM	Bukhara	Zeravshan	Echkiliksoy	40.267551	64.743333	27/07/2019
257.	Abbottina_rivularis_465	SWU28072019465	FFU252-21	MW649409	Gobionidae	*Abbottina rivularis*	Sheraliev, BM	Sheraliev, BM	Bukhara	Zeravshan	Zeravshan	40.071012	64.778429	28/07/2019
258.	Abbottina_rivularis_498	SWU31072019498	FFU253-21	MW649410	Gobionidae	*Abbottina rivularis*	Sheraliev, BM	Sheraliev, BM	Khorazm	Amu Darya	Amu Darya	41.650833	60.704722	31/07/2019
259.	Abbottina_rivularis_602	SWU18082019602	FFU254-21	MW649411	Gobionidae	*Abbottina rivularis*	Sheraliev, BM	Sheraliev, BM	Tashkent	Syr Darya	Chirchik river	41.274444	69.389166	18/08/2019
260.	Abbottina_rivularis_690	SWU27042019690	FFU255-21	MW649412	Gobionidae	*Abbottina rivularis*	Allayarov, S	Sheraliev, BM	Surxondaryo	Amu Darya	Karatag river	38.363055	68.072059	27/04/2019
261.	Abbottina_rivularis_691	SWU27042019691	FFU256-21	MW649413	Gobionidae	*Abbottina rivularis*	Allayarov, S	Sheraliev, BM	Surxondaryo	Amu Darya	Karatag river	38.363055	68.072059	27/04/2019
262.	Gobio_lepidolaemus_031	SWU31082016031	FFU257-21	MW649414	Gobionidae	*Gobio lepidolaemus*	Sheraliev, BM	Sheraliev, BM	Namangan	Syr Darya	Syr Darya	40.882777	71.442222	31/08/2016
263.	Gobio_lepidolaemus_061	SWU07012017061	FFU258-21	MW649415	Gobionidae	*Gobio lepidolaemus*	Sheraliev, BM	Sheraliev, BM	Fergana	Syr Darya	Unnamed stream	40.366437	71.316907	07/01/2017
264.	Gobio_lepidolaemus_062	SWU07012017062	FFU259-21	MW649416	Gobionidae	*Gobio lepidolaemus*	Sheraliev, BM	Sheraliev, BM	Fergana	Syr Darya	Unnamed stream	40.366437	71.316907	07/01/2017
265.	Gobio_lepidolaemus_063	SWU07012017063	FFU260-21	MW649417	Gobionidae	*Gobio lepidolaemus*	Sheraliev, BM	Sheraliev, BM	Fergana	Syr Darya	Unnamed stream	40.366437	71.316907	07/01/2017
266.	Gobio_lepidolaemus_098	SWU25012018098	FFU261-21	MW649418	Gobionidae	*Gobio lepidolaemus*	Sheraliev, BM	Sheraliev, BM	Fergana	Syr Darya	Fergana Canal	40.388497	71.327960	25/01/2018
267.	Gobio_lepidolaemus_099	SWU25012018099	FFU262-21	MW649419	Gobionidae	*Gobio lepidolaemus*	Sheraliev, BM	Sheraliev, BM	Fergana	Syr Darya	Fergana Canal	40.388497	71.327960	25/01/2018
268.	Gobio_lepidolaemus_100	SWU25012018100	FFU263-21	MW649420	Gobionidae	*Gobio lepidolaemus*	Sheraliev, BM	Sheraliev, BM	Fergana	Syr Darya	Fergana Canal	40.388497	71.327960	25/01/2018
269.	Gobio_lepidolaemus_192	SWU11082018192	FFU264-21	MW649421	Gobionidae	*Gobio lepidolaemus*	Sheraliev, BM	Sheraliev, BM	Fergana	Syr Darya	Unnamed stream	40.312769	71.811647	11/08/2018
270.	Gobio_lepidolaemus_193	SWU11082018193	FFU265-21	MW649422	Gobionidae	*Gobio lepidolaemus*	Sheraliev, BM	Sheraliev, BM	Fergana	Syr Darya	Unnamed stream	40.312769	71.811647	11/08/2018
271.	Gobio_lepidolaemus_194	SWU11082018194	FFU266-21	MW649423	Gobionidae	*Gobio lepidolaemus*	Sheraliev, BM	Sheraliev, BM	Fergana	Syr Darya	Unnamed stream	40.312769	71.811647	11/08/2018
272.	Gobio_lepidolaemus_195	SWU11082018195	FFU267-21	MW649424	Gobionidae	*Gobio lepidolaemus*	Sheraliev, BM	Sheraliev, BM	Fergana	Syr Darya	Unnamed stream	40.312769	71.811647	11/08/2018
273.	Gobio_lepidolaemus_196	SWU11082018196	FFU268-21	MW649425	Gobionidae	*Gobio lepidolaemus*	Sheraliev, BM	Sheraliev, BM	Fergana	Syr Darya	Unnamed stream	40.312769	71.811647	11/08/2018
274.	Gobio_lepidolaemus_197	SWU11082018197	FFU269-21	MW649426	Gobionidae	*Gobio lepidolaemus*	Sheraliev, BM	Sheraliev, BM	Fergana	Syr Darya	Unnamed stream	40.312769	71.811647	11/08/2018
275.	Gobio_lepidolaemus_257	SWU19072019257	FFU270-21	MN810113	Gobionidae	*Gobio lepidolaemus*	Sheraliev, BM	Sheraliev, BM	Surxondaryo	Amu Darya	Surkhan Darya	37.255833	67.338611	19/07/2019
276.	Gobio_lepidolaemus_568	SWU09082019568	FFU271-21	MN810114	Gobionidae	*Gobio lepidolaemus*	Sheraliev, BM	Sheraliev, BM	Namangan	Syr Darya	Kara Darya	40.918813	71.828896	09/08/2019
277.	Gobio_lepidolaemus_569	SWU09082019569	FFU272-21	MW649427	Gobionidae	*Gobio lepidolaemus*	Sheraliev, BM	Sheraliev, BM	Namangan	Syr Darya	Kara Darya	40.918813	71.828896	09/08/2019
278.	Gobio_lepidolaemus_579	SWU09082019579	FFU273-21	MW649428	Gobionidae	*Gobio lepidolaemus*	Sheraliev, BM	Sheraliev, BM	Namangan	Syr Darya	Kara Darya	40.918813	71.828896	09/08/2019
279.	Gobio_lepidolaemus_603	SWU18082019603	FFU274-21	MW649429	Gobionidae	*Gobio lepidolaemus*	Sheraliev, BM	Sheraliev, BM	Tashkent	Syr Darya	Chirchik river	41.274444	69.389166	18/08/2019
280.	Gobio_nigrescens_241	SWU16082016241	FFU275-21	MN810111	Gobionidae	*Gobio nigrescens*	Sheraliev, BM	Sheraliev, BM	Qashqadaryo	Amu Darya	Qorasuv Stream	38.570458	66.296166	16/08/2016
281.	Gobio_nigrescens_247	SWU16072018247	FFU276-21	MW649430	Gobionidae	*Gobio nigrescens*	Sheraliev, BM	Sheraliev, BM	Bukhara	Zeravshan	Zeravshan	40.146944	64.891666	16/07/2018
282.	Gobio_nigrescens_429	SWU27072019429	FFU277-21	MW649431	Gobionidae	*Gobio nigrescens*	Sheraliev, BM	Sheraliev, BM	Bukhara	Zeravshan	Echkiliksoy	40.267551	64.743333	27/07/2019
283.	Gobio_nigrescens_435	SWU27072019435	FFU278-21	MW649432	Gobionidae	*Gobio nigrescens*	Sheraliev, BM	Sheraliev, BM	Bukhara	Zeravshan	Echkiliksoy	40.267551	64.743333	27/07/2019
284.	Gobio_nigrescens_436	SWU27072019436	FFU279-21	MW649433	Gobionidae	*Gobio nigrescens*	Sheraliev, BM	Sheraliev, BM	Bukhara	Zeravshan	Echkiliksoy	40.267551	64.743333	27/07/2019
285.	Gobio_nigrescens_531	SWU02082019531	FFU280-21	MN810112	Gobionidae	*Gobio nigrescens*	Sheraliev, BM	Sheraliev, BM	Samarkand	Zeravshan	Zeravshan	39.676388	67.076111	02/08/2019
286.	Gobio_nigrescens_532	SWU02082019532	FFU281-21	MW649434	Gobionidae	*Gobio nigrescens*	Sheraliev, BM	Sheraliev, BM	Samarkand	Zeravshan	Zeravshan	39.676388	67.076111	02/08/2019
287.	Gobio_nigrescens_533	SWU02082019533	FFU282-21	MW649435	Gobionidae	*Gobio nigrescens*	Sheraliev, BM	Sheraliev, BM	Samarkand	Zeravshan	Zeravshan	39.676388	67.076111	02/08/2019
288.	Gobio_sibiricus_373	SWU23072019373	FFU283-21	MN810110	Gobionidae	*Gobio sibiricus*	Sheraliev, BM	Sheraliev, BM	Surxondaryo	Amu Darya	Tupalang River	38.318966	68.006169	23/07/2019
289.	Gobio_sibiricus_383	SWU23072019383	FFU284-21	MW649436	Gobionidae	*Gobio sibiricus*	Sheraliev, BM	Sheraliev, BM	Surxondaryo	Amu Darya	Tupalang River	38.318966	68.006169	23/07/2019
290.	Pseudorasbora_parva_064	SWU07012017064	FFU285-21	MW649437	Gobionidae	*Pseudorasbora parva*	Sheraliev, BM	Sheraliev, BM	Fergana	Syr Darya	Unnamed stream	40.366437	71.316907	07/01/2017
291.	Pseudorasbora_parva_065	SWU07012017065	FFU286-21	MW649438	Gobionidae	*Pseudorasbora parva*	Sheraliev, BM	Sheraliev, BM	Fergana	Syr Darya	Unnamed stream	40.366437	71.316907	07/01/2017
292.	Pseudorasbora_parva_101	SWU25012018101	FFU287-21	MW649439	Gobionidae	*Pseudorasbora parva*	Sheraliev, BM	Sheraliev, BM	Fergana	Syr Darya	Fergana Canal	40.388497	71.327960	25/01/2018
293.	Pseudorasbora_parva_102	SWU25012018102	FFU288-21	MW649440	Gobionidae	*Pseudorasbora parva*	Sheraliev, BM	Sheraliev, BM	Fergana	Syr Darya	Fergana Canal	40.388497	71.327960	25/01/2018
294.	Pseudorasbora_parva_103	SWU25012018103	FFU289-21	MW649441	Gobionidae	*Pseudorasbora parva*	Sheraliev, BM	Sheraliev, BM	Fergana	Syr Darya	Fergana Canal	40.388497	71.327960	25/01/2018
295.	Pseudorasbora_parva_144	SWU21022018144	FFU290-21	MW649442	Gobionidae	*Pseudorasbora parva*	Sheraliev, BM	Sheraliev, BM	Tashkent	Syr Darya	Chirchik River	41.370266	69.512026	21/02/2018

296.	Pseudorasbora_parva_266	SWU19072019266	FFU291-21	MW649443	Gobionidae	*Pseudorasbora parva*	Sheraliev, BM	Sheraliev, BM	Surxondaryo	Amu Darya	Surkhan Darya	37.255833	67.338611	19/07/2019
297.	Pseudorasbora_parva_309	SWU21072019309	FFU292-21	MW649444	Gobionidae	*Pseudorasbora parva*	Sheraliev, BM	Sheraliev, BM	Surxondaryo	Amu Darya	Surkhan Darya	37.238888	67.329722	21/07/2019
298.	Pseudorasbora_parva_442	SWU27072019442	FFU293-21	MW649445	Gobionidae	*Pseudorasbora parva*	Sheraliev, BM	Sheraliev, BM	Bukhara	Zeravshan	Echkiliksoy	40.267551	64.743333	27/07/2019
299.	Pseudorasbora_parva_580	SWU09082019580	FFU294-21	MW649446	Gobionidae	*Pseudorasbora parva*	Sheraliev, BM	Sheraliev, BM	Namangan	Syr Darya	Kara Darya	40.918813	71.828896	09/08/2019
300.	Pseudorasbora_parva_692	SWU27042019692	FFU295-21	MW649447	Gobionidae	*Pseudorasbora parva*	Allayarov, S	Sheraliev, BM	Surxondaryo	Amu Darya	Karatag river	38.363055	68.072059	27/04/2019
301.	Abramis_brama_180	SWU18072018180	FFU296-21	MW649448	Leuciscidae	*Abramis brama*	Sheraliev, BM	Sheraliev, BM	Navoiy	Zeravshan	Tudakul Lake	39.801388	64.772222	18/07/2018
302.	Abramis_brama_181	SWU18072018181	FFU297-21	MW649449	Leuciscidae	*Abramis brama*	Sheraliev, BM	Sheraliev, BM	Navoiy	Zeravshan	Tudakul Lake	39.801388	64.772222	18/07/2018
303.	Alburnoides_holciki_001	SWU08082016001	FFU298-21	MN872408	Leuciscidae	*Alburnoides holciki*	Sheraliev, BM	Sheraliev, BM	Surxondaryo	Amu Darya	Tupalang river	38.399802	67.944766	08/08/2016
304.	Alburnoides_holciki_002	SWU08082016002	FFU299-21	MW649450	Leuciscidae	*Alburnoides holciki*	Sheraliev, BM	Sheraliev, BM	Surxondaryo	Amu Darya	Tupalang river	38.399802	67.944766	08/08/2016
305.	Alburnoides_holciki_003	SWU08082016003	FFU300-21	MW649451	Leuciscidae	*Alburnoides holciki*	Sheraliev, BM	Sheraliev, BM	Surxondaryo	Amu Darya	Tupalang river	38.399802	67.944766	08/08/2016
306.	Alburnoides_holciki_155	SWU16072018155	FFU301-21	MW649452	Leuciscidae	*Alburnoides holciki*	Sheraliev, BM	Sheraliev, BM	Bukhara	Zeravshan	Zeravshan	40.146944	64.891666	16/07/2018
307.	Alburnoides_holciki_156	SWU16072018156	FFU302-21	MW649453	Leuciscidae	*Alburnoides holciki*	Sheraliev, BM	Sheraliev, BM	Bukhara	Zeravshan	Zeravshan	40.146944	64.891666	16/07/2018
308.	Alburnoides_holciki_157	SWU16072018157	FFU303-21	MW649454	Leuciscidae	*Alburnoides holciki*	Sheraliev, BM	Sheraliev, BM	Bukhara	Zeravshan	Zeravshan	40.146944	64.891666	16/07/2018
309.	Alburnoides_holciki_158	SWU16072018158	FFU304-21	MW649455	Leuciscidae	*Alburnoides holciki*	Sheraliev, BM	Sheraliev, BM	Bukhara	Zeravshan	Zeravshan	40.146944	64.891666	16/07/2018
310.	Alburnoides_holciki_159	SWU16072018159	FFU305-21	MW649456	Leuciscidae	*Alburnoides holciki*	Sheraliev, BM	Sheraliev, BM	Bukhara	Zeravshan	Zeravshan	40.146944	64.891666	16/07/2018
311.	Alburnoides_holciki_236	SWU12082016236	FFU306-21	MW649457	Leuciscidae	*Alburnoides holciki*	Sheraliev, BM	Sheraliev, BM	Surxondaryo	Amu Darya	Tupalang	38.376388	67.963166	12/08/2016
312.	Alburnoides_holciki_237	SWU12082016237	FFU307-21	MW649458	Leuciscidae	*Alburnoides holciki*	Sheraliev, BM	Sheraliev, BM	Surxondaryo	Amu Darya	Tupalang	38.376388	67.963166	12/08/2016
313.	Alburnoides_holciki_238	SWU12082016238	FFU308-21	MW649459	Leuciscidae	*Alburnoides holciki*	Sheraliev, BM	Sheraliev, BM	Surxondaryo	Amu Darya	Tupalang	38.376388	67.963166	12/08/2016
314.	Alburnoides_holciki_250	SWU16072018250	FFU309-21	MW649460	Leuciscidae	*Alburnoides holciki*	Sheraliev, BM	Sheraliev, BM	Bukhara	Zeravshan	Zeravshan	40.146944	64.891666	16/07/2018
315.	Alburnoides_holciki_251	SWU16072018251	FFU310-21	MW649461	Leuciscidae	*Alburnoides holciki*	Sheraliev, BM	Sheraliev, BM	Bukhara	Zeravshan	Zeravshan	40.146944	64.891666	16/07/2018
316.	Alburnoides_holciki_267	SWU19072019267	FFU311-21	MW649462	Leuciscidae	*Alburnoides holciki*	Sheraliev, BM	Sheraliev, BM	Surxondaryo	Amu Darya	Surkhan Darya	37.255833	67.338611	19/07/2019
317.	Alburnoides_holciki_268	SWU19072019268	FFU312-21	MW649463	Leuciscidae	*Alburnoides holciki*	Sheraliev, BM	Sheraliev, BM	Surxondaryo	Amu Darya	Surkhan Darya	37.255833	67.338611	19/07/2019
318.	Alburnoides_holciki_269	SWU19072019269	FFU313-21	MW649464	Leuciscidae	*Alburnoides holciki*	Sheraliev, BM	Sheraliev, BM	Surxondaryo	Amu Darya	Surkhan Darya	37.255833	67.338611	19/07/2019
319.	Alburnoides_holciki_270	SWU19072019270	FFU314-21	MW649465	Leuciscidae	*Alburnoides holciki*	Sheraliev, BM	Sheraliev, BM	Surxondaryo	Amu Darya	Surkhan Darya	37.255833	67.338611	19/07/2019
320.	Alburnoides_holciki_275	SWU19072019275	FFU315-21	MW649466	Leuciscidae	*Alburnoides holciki*	Sheraliev, BM	Sheraliev, BM	Surxondaryo	Amu Darya	Surkhan Darya	37.255833	67.338611	19/07/2019
321.	Alburnoides_holciki_276	SWU19072019276	FFU316-21	MW649467	Leuciscidae	*Alburnoides holciki*	Sheraliev, BM	Sheraliev, BM	Surxondaryo	Amu Darya	Surkhan Darya	37.255833	67.338611	19/07/2019
322.	Alburnoides_holciki_277	SWU19072019277	FFU317-21	MW649468	Leuciscidae	*Alburnoides holciki*	Sheraliev, BM	Sheraliev, BM	Surxondaryo	Amu Darya	Surkhan Darya	37.255833	67.338611	19/07/2019
323.	Alburnoides_holciki_278	SWU19072019278	FFU318-21	MW649469	Leuciscidae	*Alburnoides holciki*	Sheraliev, BM	Sheraliev, BM	Surxondaryo	Amu Darya	Surkhan Darya	37.255833	67.338611	19/07/2019
324.	Alburnoides_holciki_279	SWU19072019279	FFU319-21	MW649470	Leuciscidae	*Alburnoides holciki*	Sheraliev, BM	Sheraliev, BM	Surxondaryo	Amu Darya	Surkhan Darya	37.255833	67.338611	19/07/2019
325.	Alburnoides_holciki_321	SWU21072019321	FFU320-21	MW649471	Leuciscidae	*Alburnoides holciki*	Sheraliev, BM	Sheraliev, BM	Surxondaryo	Amu Darya	Surkhan Darya	37.238888	67.329722	21/07/2019
326.	Alburnoides_holciki_323	SWU21072019323	FFU321-21	MW649472	Leuciscidae	*Alburnoides holciki*	Sheraliev, BM	Sheraliev, BM	Surxondaryo	Amu Darya	Surkhan Darya	37.238888	67.329722	21/07/2019
327.	Alburnoides_holciki_369	SWU19072019369	FFU322-21	MW649473	Leuciscidae	*Alburnoides holciki*	Sheraliev, BM	Sheraliev, BM	Surxondaryo	Amu Darya	Surkhan Darya	37.255833	67.338611	19/07/2019
328.	Alburnoides_holciki_370	SWU19072019370	FFU323-21	MW649474	Leuciscidae	*Alburnoides holciki*	Sheraliev, BM	Sheraliev, BM	Surxondaryo	Amu Darya	Surkhan Darya	37.255833	67.338611	19/07/2019
329.	Alburnoides_holciki_409	SWU25072019409	FFU324-21	MW649475	Leuciscidae	*Alburnoides holciki*	Sheraliev, BM	Sheraliev, BM	Surxondaryo	Amu Darya	Sherobod river	37.747222	66.996666	25/07/2019
330.	Alburnoides_holciki_410	SWU25072019410	FFU325-21	MW649476	Leuciscidae	*Alburnoides holciki*	Sheraliev, BM	Sheraliev, BM	Surxondaryo	Amu Darya	Sherobod river	37.747222	66.996666	25/07/2019
331.	Alburnoides_holciki_411	SWU25072019411	FFU326-21	MW649477	Leuciscidae	*Alburnoides holciki*	Sheraliev, BM	Sheraliev, BM	Surxondaryo	Amu Darya	Sherobod river	37.747222	66.996666	25/07/2019
332.	Alburnoides_holciki_426	SWU27072019426	FFU327-21	MW649478	Leuciscidae	*Alburnoides holciki*	Sheraliev, BM	Sheraliev, BM	Bukhara	Zeravshan	Echkiliksoy	40.267551	64.743333	27/07/2019
333.	Alburnoides_holciki_427	SWU27072019427	FFU328-21	MW649479	Leuciscidae	*Alburnoides holciki*	Sheraliev, BM	Sheraliev, BM	Bukhara	Zeravshan	Echkiliksoy	40.267551	64.743333	27/07/2019
334.	Alburnoides_holciki_443	SWU27072019443	FFU329-21	MW649480	Leuciscidae	*Alburnoides holciki*	Sheraliev, BM	Sheraliev, BM	Bukhara	Zeravshan	Echkiliksoy	40.267551	64.743333	27/07/2019
335.	Alburnoides_holciki_444	SWU27072019444	FFU330-21	MW649481	Leuciscidae	*Alburnoides holciki*	Sheraliev, BM	Sheraliev, BM	Bukhara	Zeravshan	Echkiliksoy	40.267551	64.743333	27/07/2019
336.	Alburnoides_holciki_445	SWU27072019445	FFU331-21	MW649482	Leuciscidae	*Alburnoides holciki*	Sheraliev, BM	Sheraliev, BM	Bukhara	Zeravshan	Echkiliksoy	40.267551	64.743333	27/07/2019
337.	Alburnoides_holciki_489	SWU31072019489	FFU332-21	MW649483	Leuciscidae	*Alburnoides holciki*	Sheraliev, BM	Sheraliev, BM	Khorazm	Amu Darya	Amu Darya	41.650833	60.704722	31/07/2019
338.	Alburnoides_holciki_536	SWU02082019536	FFU333-21	MW649484	Leuciscidae	*Alburnoides holciki*	Sheraliev, BM	Sheraliev, BM	Samarkand	Zeravshan	Zeravshan	39.676388	67.076111	02/08/2019
339.	Alburnoides_holciki_537	SWU02082019537	FFU334-21	MW649485	Leuciscidae	*Alburnoides holciki*	Sheraliev, BM	Sheraliev, BM	Samarkand	Zeravshan	Zeravshan	39.676388	67.076111	02/08/2019
340.	Alburnoides_holciki_538	SWU02082019538	FFU335-21	MW649486	Leuciscidae	*Alburnoides holciki*	Sheraliev, BM	Sheraliev, BM	Samarkand	Zeravshan	Zeravshan	39.676388	67.076111	02/08/2019
341.	Alburnoides_holciki_697	SWU27042019697	FFU336-21	MW649487	Leuciscidae	*Alburnoides holciki*	Allayarov, S	Sheraliev, BM	Surxondaryo	Amu Darya	Karatag river	38.363055	68.072059	27/04/2019
342.	Alburnoides_holciki_698	SWU27042019698	FFU337-21	MW649488	Leuciscidae	*Alburnoides holciki*	Allayarov, S	Sheraliev, BM	Surxondaryo	Amu Darya	Karatag river	38.363055	68.072059	27/04/2019
343.	Alburnoides_holciki_699	SWU27042019699	FFU338-21	MW649489	Leuciscidae	*Alburnoides holciki*	Allayarov, S	Sheraliev, BM	Surxondaryo	Amu Darya	Karatag river	38.363055	68.072059	27/04/2019
344.	Alburnoides_taeniatus_430	SWU27072019430	FFU339-21	MW649490	Leuciscidae	*Alburnus taeniatus*	Sheraliev, BM	Sheraliev, BM	Bukhara	Zeravshan	Echkiliksoy	40.267551	64.743333	27/07/2019
345.	Alburnoides_taeniatus_534	SWU02082019534	FFU340-21	MW649491	Leuciscidae	*Alburnus taeniatus*	Sheraliev, BM	Sheraliev, BM	Samarkand	Zeravshan	Zeravshan	39.676388	67.076111	02/08/2019
346.	Alburnoides_taeniatus_535	SWU02082019535	FFU341-21	MW649492	Leuciscidae	*Alburnus taeniatus*	Sheraliev, BM	Sheraliev, BM	Samarkand	Zeravshan	Zeravshan	39.676388	67.076111	02/08/2019
347.	Alburnus_chalcoides_016	SWU15082016016	FFU342-21	MW649493	Leuciscidae	*Alburnus chalcoides*	Sheraliev, BM	Sheraliev, BM	Qashqadaryo	Amu Darya	Qorasuv Stream	38.570458	66.296166	15/08/2016
348.	Alburnus_chalcoides_017	SWU15082016017	FFU343-21	MW649494	Leuciscidae	*Alburnus chalcoides*	Sheraliev, BM	Sheraliev, BM	Qashqadaryo	Amu Darya	Qorasuv Stream	38.570458	66.296166	15/08/2016
349.	Alburnus_chalcoides_018	SWU15082016018	FFU344-21	MW649495	Leuciscidae	*Alburnus chalcoides*	Sheraliev, BM	Sheraliev, BM	Qashqadaryo	Amu Darya	Qorasuv Stream	38.570458	66.296166	15/08/2016
350.	Alburnus_chalcoides_177	SWU18072018177	FFU345-21	MW649496	Leuciscidae	*Alburnus chalcoides*	Sheraliev, BM	Sheraliev, BM	Navoiy	Zeravshan	Tudakul Lake	39.801388	64.772222	18/07/2018
351.	Alburnus_chalcoides_274	SWU19072019274	FFU346-21	MW649497	Leuciscidae	*Alburnus chalcoides*	Sheraliev, BM	Sheraliev, BM	Surxondaryo	Amu Darya	Surkhan Darya	37.255833	67.338611	19/07/2019
352.	Alburnus_chalcoides_280	SWU19072019280	FFU347-21	MW649498	Leuciscidae	*Alburnus chalcoides*	Sheraliev, BM	Sheraliev, BM	Surxondaryo	Amu Darya	Surkhan Darya	37.255833	67.338611	19/07/2019
353.	Alburnus_chalcoides_431	SWU27072019431	FFU348-21	MW649499	Leuciscidae	*Alburnus chalcoides*	Sheraliev, BM	Sheraliev, BM	Bukhara	Zeravshan	Echkiliksoy	40.267551	64.743333	27/07/2019
354.	Alburnus_chalcoides_440	SWU27072019440	FFU349-21	MW649500	Leuciscidae	*Alburnus chalcoides*	Sheraliev, BM	Sheraliev, BM	Bukhara	Zeravshan	Echkiliksoy	40.267551	64.743333	27/07/2019
355.	Alburnus_chalcoides_441	SWU27072019441	FFU350-21	MW649501	Leuciscidae	*Alburnus chalcoides*	Sheraliev, BM	Sheraliev, BM	Bukhara	Zeravshan	Echkiliksoy	40.267551	64.743333	27/07/2019
356.	Alburnus_chalcoides_471	SWU29072019471	FFU351-21	MW649502	Leuciscidae	*Alburnus chalcoides*	Sheraliev, BM	Sheraliev, BM	Bukhara	Zeravshan	Unnamed stream	40.147222	64.905805	29/07/2019
357.	Alburnus_chalcoides_472	SWU29072019472	FFU352-21	MW649503	Leuciscidae	*Alburnus chalcoides*	Sheraliev, BM	Sheraliev, BM	Bukhara	Zeravshan	Unnamed stream	40.147222	64.905805	29/07/2019
358.	Alburnus_chalcoides_473	SWU29072019473	FFU353-21	MW649504	Leuciscidae	*Alburnus chalcoides*	Sheraliev, BM	Sheraliev, BM	Bukhara	Zeravshan	Unnamed stream	40.147222	64.905805	29/07/2019
359.	Alburnus_oblongus_591	SWU18082019591	FFU354-21	MW649505	Leuciscidae	*Alburnus oblongus*	Sheraliev, BM	Sheraliev, BM	Tashkent	Syr Darya	Chirchik river	41.274444	69.389166	18/08/2019
360.	Alburnus_oblongus_592	SWU18082019592	FFU355-21	MW649506	Leuciscidae	*Alburnus oblongus*	Sheraliev, BM	Sheraliev, BM	Tashkent	Syr Darya	Chirchik river	41.274444	69.389166	18/08/2019
361.	Alburnus_oblongus_593	SWU18082019593	FFU356-21	MW649507	Leuciscidae	*Alburnus oblongus*	Sheraliev, BM	Sheraliev, BM	Tashkent	Syr Darya	Chirchik river	41.274444	69.389166	18/08/2019
362.	Alburnus_oblongus_594	SWU18082019594	FFU357-21	MW649508	Leuciscidae	*Alburnus oblongus*	Sheraliev, BM	Sheraliev, BM	Tashkent	Syr Darya	Chirchik river	41.274444	69.389166	18/08/2019
363.	Alburnus_oblongus_604	SWU18082019604	FFU358-21	MW649509	Leuciscidae	*Alburnus oblongus*	Sheraliev, BM	Sheraliev, BM	Tashkent	Syr Darya	Chirchik river	41.450555	69.598611	18/08/2019
364.	Alburnus_oblongus_605	SWU18082019605	FFU359-21	MW649510	Leuciscidae	*Alburnus oblongus*	Sheraliev, BM	Sheraliev, BM	Tashkent	Syr Darya	Chirchik river	41.450555	69.598611	18/08/2019
365.	Alburnus_oblongus_606	SWU18082019606	FFU360-21	MW649511	Leuciscidae	*Alburnus oblongus*	Sheraliev, BM	Sheraliev, BM	Tashkent	Syr Darya	Chirchik river	41.450555	69.598611	18/08/2019
366.	Alburnus_oblongus_607	SWU18082019607	FFU361-21	MW649512	Leuciscidae	*Alburnus oblongus*	Sheraliev, BM	Sheraliev, BM	Tashkent	Syr Darya	Chirchik river	41.450555	69.598611	18/08/2019
367.	Capoetobrama_kuschakewitschi_314	SWU21072019313	FFU362-21	MW649513	Leuciscidae	*Capoetobrama kuschakewitschi*	Allayarov, S	Sheraliev, BM	Surxondaryo	Amu Darya	Amu Darya	37.235555	67.660555	21/07/2019
368.	Capoetobrama_kuschakewitschi_315	SWU21072019313	FFU363-21	MW649514	Leuciscidae	*Capoetobrama kuschakewitschi*	Allayarov, S	Sheraliev, BM	Surxondaryo	Amu Darya	Amu Darya	37.235555	67.660555	21/07/2019
369.	Capoetobrama_kuschakewitschi_316	SWU21072019313	FFU364-21	MW649515	Leuciscidae	*Capoetobrama kuschakewitschi*	Allayarov, S	Sheraliev, BM	Surxondaryo	Amu Darya	Amu Darya	37.235555	67.660555	21/07/2019

370.	Capoetobrama_kuschakewitschi_317	SWU21072019313	FFU365-21	MW649516	Leuciscidae	*Capoetobrama kuschakewitschi*	Allayarov, S	Sheraliev, BM	Surxondaryo	Amu Darya	Amu Darya	37.235555	67.660555	21/07/2019
371.	Capoetobrama_kuschakewitschi_491	SWU31072019491	FFU366-21	MW649517	Leuciscidae	*Capoetobrama kuschakewitschi*	Sheraliev, BM	Sheraliev, BM	Khorazm	Amu Darya	Amu Darya	41.650833	60.704722	31/07/2019
372.	Capoetobrama_kuschakewitschi_492	SWU31072019492	FFU367-21	MW649518	Leuciscidae	*Capoetobrama kuschakewitschi*	Sheraliev, BM	Sheraliev, BM	Khorazm	Amu Darya	Amu Darya	41.650833	60.704722	31/07/2019
373.	Capoetobrama_kuschakewitschi_499	SWU31072019499	FFU368-21	MW649519	Leuciscidae	*Capoetobrama kuschakewitschi*	Sheraliev, BM	Sheraliev, BM	Khorazm	Amu Darya	Amu Darya	41.650833	60.704722	31/07/2019
374.	Capoetobrama_kuschakewitschi_501	SWU31072019501	FFU369-21	MW649520	Leuciscidae	*Capoetobrama kuschakewitschi*	Sheraliev, BM	Sheraliev, BM	Khorazm	Amu Darya	Amu Darya	41.650833	60.704722	31/07/2019
375.	Leuciscus_aspius_673	SWU21082019673	FFU370-21	MW649521	Leuciscidae	*Leuciscus aspius*	Rozimov, A	Sheraliev, BM	Khorazm	Amu Darya	Amu Darya	41.948638	60.444194	21/08/2019
376.	Leuciscus_lehmanni_015	SWU15082016015	FFU371-21	MN872388	Leuciscidae	*Leuciscus lehmanni*	Sheraliev, BM	Sheraliev, BM	Qashqadaryo	Amu Darya	Qorasuv Stream	38.570458	66.296166	15/08/2016
377.	Leuciscus_lehmanni_133	SWU22022018133	FFU372-21	MN872389	Leuciscidae	*Leuciscus lehmanni*	Sheraliev, BM	Sheraliev, BM	Bukhara	Zeravshan	Qoraqir Lake	40.385644	63.308388	22/02/2018
378.	Leuciscus_lehmanni_271	SWU19072019271	FFU373-21	MN872390	Leuciscidae	*Leuciscus lehmanni*	Sheraliev, BM	Sheraliev, BM	Surxondaryo	Amu Darya	Surkhan Darya	37.255833	67.338611	19/07/2019
379.	Leuciscus_lehmanni_272	SWU19072019271	FFU374-21	MN872391	Leuciscidae	*Leuciscus lehmanni*	Sheraliev, BM	Sheraliev, BM	Surxondaryo	Amu Darya	Surkhan Darya	37.255833	67.338611	19/07/2019
380.	Leuciscus_lehmanni_273	SWU19072019271	FFU375-21	MN872392	Leuciscidae	*Leuciscus lehmanni*	Sheraliev, BM	Sheraliev, BM	Surxondaryo	Amu Darya	Surkhan Darya	37.255833	67.338611	19/07/2019
381.	Leuciscus_lehmanni_295	SWU21072019295	FFU376-21	MN872393	Leuciscidae	*Leuciscus lehmanni*	Sheraliev, BM	Sheraliev, BM	Surxondaryo	Amu Darya	Surkhan Darya	37.238888	67.329722	21/07/2019
382.	Leuciscus_lehmanni_304	SWU21072019304	FFU377-21	MN872394	Leuciscidae	*Leuciscus lehmanni*	Sheraliev, BM	Sheraliev, BM	Surxondaryo	Amu Darya	Surkhan Darya	37.238888	67.329722	21/07/2019
383.	Leuciscus_lehmanni_305	SWU21072019305	FFU378-21	MN872395	Leuciscidae	*Leuciscus lehmanni*	Sheraliev, BM	Sheraliev, BM	Surxondaryo	Amu Darya	Surkhan Darya	37.238888	67.329722	21/07/2019
384.	Leuciscus_lehmanni_475	SWU29072019475	FFU379-21	MN872396	Leuciscidae	*Leuciscus lehmanni*	Sheraliev, BM	Sheraliev, BM	Bukhara	Zeravshan	Unnamed stream	40.147222	64.905805	29/07/2019
385.	Leuciscus_lehmanni_476	SWU29072019475	FFU380-21	MN872397	Leuciscidae	*Leuciscus lehmanni*	Sheraliev, BM	Sheraliev, BM	Bukhara	Zeravshan	Unnamed stream	40.147222	64.905805	29/07/2019
386.	Pelecus_cultratus_036	SWU31082016036	FFU381-21	MW649522	Leuciscidae	*Pelecus cultratus*	Sheraliev, BM	Sheraliev, BM	Namangan	Syr Darya	Syr Darya	40.882777	71.442222	31/08/2016
387.	Pelecus_cultratus_037	SWU31082016037	FFU382-21	MW649523	Leuciscidae	*Pelecus cultratus*	Sheraliev, BM	Sheraliev, BM	Namangan	Syr Darya	Syr Darya	40.882777	71.442222	31/08/2016
388.	Pelecus_cultratus_038	SWU31082016038	FFU383-21	MW649524	Leuciscidae	*Pelecus cultratus*	Sheraliev, BM	Sheraliev, BM	Namangan	Syr Darya	Syr Darya	40.882777	71.442222	31/08/2016
389.	Pelecus_cultratus_039	SWU31082016039	FFU384-21	MW649525	Leuciscidae	*Pelecus cultratus*	Sheraliev, BM	Sheraliev, BM	Namangan	Syr Darya	Syr Darya	40.882777	71.442222	31/08/2016
390.	Petroleuciscus_squaliusculus_068	SWU15012017068	FFU385-21	MN872398	Leuciscidae	*Petroleuciscus squaliusculus*	Sheraliev, BM	Sheraliev, BM	Fergana	Syr Darya	Unnamed stream	40.326408	71.820508	15/01/2017
391.	Petroleuciscus_squaliusculus_069	SWU15012017069	FFU386-21	MN872399	Leuciscidae	*Petroleuciscus squaliusculus*	Sheraliev, BM	Sheraliev, BM	Fergana	Syr Darya	Unnamed stream	40.326408	71.820508	15/01/2017
392.	Petroleuciscus_squaliusculus_070	SWU15012017070	FFU387-21	MN872400	Leuciscidae	*Petroleuciscus squaliusculus*	Sheraliev, BM	Sheraliev, BM	Fergana	Syr Darya	Unnamed stream	40.326408	71.820508	15/01/2017
393.	Petroleuciscus_squaliusculus_128	SWU08022018128	FFU388-21	MN872401	Leuciscidae	*Petroleuciscus squaliusculus*	Sheraliev, BM	Sheraliev, BM	Fergana	Syr Darya	Sokh River	40.340176	71.013559	08/02/2018
394.	Petroleuciscus_squaliusculus_191	SWU11082018191	FFU389-21	MN872402	Leuciscidae	*Petroleuciscus squaliusculus*	Sheraliev, BM	Sheraliev, BM	Fergana	Syr Darya	Unnamed stream	40.312769	71.811647	11/08/2018
395.	Petroleuciscus_squaliusculus_581	SWU09082019581	FFU390-21	MN872403	Leuciscidae	*Petroleuciscus squaliusculus*	Sheraliev, BM	Sheraliev, BM	Namangan	Syr Darya	Naryn River	40.940911	71.853631	09/08/2019
396.	Petroleuciscus_squaliusculus_582	SWU09082019582	FFU391-21	MN872404	Leuciscidae	*Petroleuciscus squaliusculus*	Sheraliev, BM	Sheraliev, BM	Namangan	Syr Darya	Naryn River	40.940911	71.853631	09/08/2019
397.	Petroleuciscus_squaliusculus_639	SWU20082019639	FFU392-21	MN872405	Leuciscidae	*Petroleuciscus squaliusculus*	Sheraliev, BM	Sheraliev, BM	Fergana	Syr Darya	Unnamed stream	40.305260	71.800688	20/08/2019
398.	Petroleuciscus_squaliusculus_640	SWU20082019640	FFU393-21	MN872406	Leuciscidae	*Petroleuciscus squaliusculus*	Sheraliev, BM	Sheraliev, BM	Fergana	Syr Darya	Unnamed stream	40.305260	71.800688	20/08/2019
399.	Petroleuciscus_squaliusculus_641	SWU20082019641	FFU394-21	MN872407	Leuciscidae	*Petroleuciscus squaliusculus*	Sheraliev, BM	Sheraliev, BM	Fergana	Syr Darya	Unnamed stream	40.305260	71.800688	20/08/2019
400.	Rutilus_rutilus_aralensis_071	SWU10072017071	FFU395-21	MW649526	Leuciscidae	*Rutilus lacustris*	Sheraliev, BM	Sheraliev, BM	Fergana	Syr Darya	Unnamed stream	40.588943	71.537333	10/07/2017
401.	Rutilus_rutilus_aralensis_072	SWU10072017072	FFU396-21	MW649527	Leuciscidae	*Rutilus lacustris*	Sheraliev, BM	Sheraliev, BM	Fergana	Syr Darya	Unnamed stream	40.588943	71.537333	10/07/2017
402.	Rutilus_rutilus_aralensis_073	SWU10072017073	FFU397-21	MW649528	Leuciscidae	*Rutilus lacustris*	Sheraliev, BM	Sheraliev, BM	Fergana	Syr Darya	Unnamed stream	40.588943	71.537333	10/07/2017
403.	Rutilus_rutilus_aralensis_074	SWU10072017074	FFU398-21	MW649529	Leuciscidae	*Rutilus lacustris*	Sheraliev, BM	Sheraliev, BM	Fergana	Syr Darya	Unnamed stream	40.588943	71.537333	10/07/2017
404.	Rutilus_rutilus_aralensis_075	SWU10072017075	FFU399-21	MW649530	Leuciscidae	*Rutilus lacustris*	Sheraliev, BM	Sheraliev, BM	Fergana	Syr Darya	Unnamed stream	40.588943	71.537333	10/07/2017
405.	Rutilus_rutilus_aralensis_076	SWU10072017076	FFU400-21	MW649531	Leuciscidae	*Rutilus lacustris*	Sheraliev, BM	Sheraliev, BM	Fergana	Syr Darya	Unnamed stream	40.588943	71.537333	10/07/2017
406.	Rutilus_rutilus_aralensis_127	SWU08022018127	FFU401-21	MW649532	Leuciscidae	*Rutilus lacustris*	Sheraliev, BM	Sheraliev, BM	Fergana	Syr Darya	Sokh River	40.340176	71.013559	08/02/2018
407.	Rutilus_rutilus_aralensis_447	SWU28072019447	FFU402-21	MW649533	Leuciscidae	*Rutilus lacustris*	Sheraliev, BM	Sheraliev, BM	Bukhara	Zeravshan	Zeravshan	40.071012	64.778429	28/07/2019
408.	Rutilus_rutilus_aralensis_448	SWU28072019448	FFU403-21	MW649534	Leuciscidae	*Rutilus lacustris*	Sheraliev, BM	Sheraliev, BM	Bukhara	Zeravshan	Zeravshan	40.071012	64.778429	28/07/2019
409.	Rutilus_rutilus_aralensis_449	SWU28072019449	FFU404-21	MW649535	Leuciscidae	*Rutilus lacustris*	Sheraliev, BM	Sheraliev, BM	Bukhara	Zeravshan	Zeravshan	40.071012	64.778429	28/07/2019
410.	Rutilus_rutilus_aralensis_674	SWU21082019674	FFU405-21	MW649536	Leuciscidae	*Rutilus lacustris*	Rozimov, A	Sheraliev, BM	Khorazm	Amu Darya	Amu Darya	41.948638	60.444194	21/08/2019
411.	Dzihunia_amudarjensis_286	SWU20072019286	FFU406-21	MW649537	Nemacheilidae	*Dzihunia amudarjensis*	Sheraliev, BM	Sheraliev, BM	Surxondaryo	Amu Darya	Amu Darya	37.235555	67.660555	20/07/2019
412.	Dzihunia_amudarjensis_287	SWU20072019287	FFU407-21	MW649538	Nemacheilidae	*Dzihunia amudarjensis*	Sheraliev, BM	Sheraliev, BM	Surxondaryo	Amu Darya	Amu Darya	37.235555	67.660555	20/07/2019
413.	Dzihunia_amudarjensis_288	SWU20072019288	FFU408-21	MW649539	Nemacheilidae	*Dzihunia amudarjensis*	Sheraliev, BM	Sheraliev, BM	Surxondaryo	Amu Darya	Amu Darya	37.235555	67.660555	20/07/2019
414.	Dzihunia_amudarjensis_289	SWU20072019289	FFU409-21	MW649540	Nemacheilidae	*Dzihunia amudarjensis*	Sheraliev, BM	Sheraliev, BM	Surxondaryo	Amu Darya	Amu Darya	37.235555	67.660555	20/07/2019
415.	Dzihunia_amudarjensis_290	SWU20072019290	FFU410-21	MW649541	Nemacheilidae	*Dzihunia amudarjensis*	Sheraliev, BM	Sheraliev, BM	Surxondaryo	Amu Darya	Amu Darya	37.235555	67.660555	20/07/2019
416.	Dzihunia_amudarjensis_291	SWU20072019291	FFU411-21	MW649542	Nemacheilidae	*Dzihunia amudarjensis*	Sheraliev, BM	Sheraliev, BM	Surxondaryo	Amu Darya	Amu Darya	37.235555	67.660555	20/07/2019
417.	Dzihunia_amudarjensis_292	SWU20072019292	FFU412-21	MW649543	Nemacheilidae	*Dzihunia amudarjensis*	Sheraliev, BM	Sheraliev, BM	Surxondaryo	Amu Darya	Amu Darya	37.235555	67.660555	20/07/2019
418.	Dzihunia_amudarjensis_293	SWU20072019293	FFU413-21	MW649544	Nemacheilidae	*Dzihunia amudarjensis*	Sheraliev, BM	Sheraliev, BM	Surxondaryo	Amu Darya	Amu Darya	37.235555	67.660555	20/07/2019
419.	Dzihunia_amudarjensis_294	SWU20072019294	FFU414-21	MW649545	Nemacheilidae	*Dzihunia amudarjensis*	Sheraliev, BM	Sheraliev, BM	Surxondaryo	Amu Darya	Amu Darya	37.235555	67.660555	20/07/2019
420.	Dzihunia_amudarjensis_669	SWU21082019669	FFU415-21	MW649546	Nemacheilidae	*Dzihunia amudarjensis*	Rozimov, A	Sheraliev, BM	Khorazm	Amu Darya	Turangasaka Ch.	41.734111	60.547944	21/08/2019
421.	Dzihunia_amudarjensis_700	SWU27042019700	FFU416-21	MW649547	Nemacheilidae	*Dzihunia amudarjensis*	Allayarov, S	Sheraliev, BM	Surxondaryo	Amu Darya	Karatag river	38.363055	68.072059	27/04/2019
422.	Dzihunia_sp1_Karatag_359	SWU22072019359	FFU603-21	MW649548	Nemacheilidae	*Dzihunia* sp1	Sheraliev, BM	Sheraliev, BM	Surxondaryo	Amu Darya	Karatag river	38.363055	68.072059	22/07/2019
423.	Dzihunia_sp1_Karatag_360	SWU22072019360	FFU632-21	MW649549	Nemacheilidae	*Dzihunia* sp1	Sheraliev, BM	Sheraliev, BM	Surxondaryo	Amu Darya	Karatag river	38.363055	68.072059	22/07/2019
424.	Dzihunia_sp1_Karatag_361	SWU22072019361	FFU633-21	MW649550	Nemacheilidae	*Dzihunia* sp1	Sheraliev, BM	Sheraliev, BM	Surxondaryo	Amu Darya	Karatag river	38.363055	68.072059	22/07/2019
425.	Dzihunia_sp1_Karatag_363	SWU22072019363	FFU634-21	MW649551	Nemacheilidae	*Dzihunia* sp1	Sheraliev, BM	Sheraliev, BM	Surxondaryo	Amu Darya	Karatag river	38.363055	68.072059	22/07/2019
426.	Dzihunia_sp1_Karatag_364	SWU22072019364	FFU635-21	MW649552	Nemacheilidae	*Dzihunia* sp1	Sheraliev, BM	Sheraliev, BM	Surxondaryo	Amu Darya	Karatag river	38.363055	68.072059	22/07/2019
427.	Dzihunia_sp1_Karatag_365	SWU22072019365	FFU636-21	MW649553	Nemacheilidae	*Dzihunia* sp1	Sheraliev, BM	Sheraliev, BM	Surxondaryo	Amu Darya	Karatag river	38.363055	68.072059	22/07/2019
428.	Dzihunia_sp1_Karatag_366	SWU22072019366	FFU637-21	MW649554	Nemacheilidae	*Dzihunia* sp1	Sheraliev, BM	Sheraliev, BM	Surxondaryo	Amu Darya	Karatag river	38.363055	68.072059	22/07/2019
429.	Dzihunia_sp1_Karatag_367	SWU22072019367	FFU638-21	MW649555	Nemacheilidae	*Dzihunia* sp1	Sheraliev, BM	Sheraliev, BM	Surxondaryo	Amu Darya	Karatag river	38.363055	68.072059	22/07/2019
430.	Dzihunia_sp1_Karatag_368	SWU22072019368	FFU639-21	MW649556	Nemacheilidae	*Dzihunia* sp1	Sheraliev, BM	Sheraliev, BM	Surxondaryo	Amu Darya	Karatag river	38.363055	68.072059	22/07/2019
431.	Dzihunia_sp1_Tupalang_377	SWU23072019377	FFU640-21	MW649557	Nemacheilidae	*Dzihunia* sp1	Sheraliev, BM	Sheraliev, BM	Surxondaryo	Amu Darya	Tupalang River	38.318966	68.006169	23/07/2019
432.	Dzihunia_sp1_Tupalang_378	SWU23072019378	FFU641-21	MW649558	Nemacheilidae	*Dzihunia* sp1	Sheraliev, BM	Sheraliev, BM	Surxondaryo	Amu Darya	Tupalang River	38.318966	68.006169	23/07/2019
433.	Dzihunia_sp2_Karatag_362	SWU22072019672	FFU642-21	MW649559	Nemacheilidae	*Dzihunia* sp2	Sheraliev, BM	Sheraliev, BM	Surxondaryo	Amu Darya	Karatag river	38.363055	68.072059	22/07/2019
434.	Dzihunia_sp2_Sherobod_405	SWU25072019405	FFU643-21	MW649560	Nemacheilidae	*Dzihunia* sp2	Sheraliev, BM	Sheraliev, BM	Surxondaryo	Amu Darya	Sherobod river	37.747222	66.996666	25/07/2019
435.	Dzihunia_sp2_Sherobod_406	SWU25072019406	FFU644-21	MW649561	Nemacheilidae	*Dzihunia* sp2	Sheraliev, BM	Sheraliev, BM	Surxondaryo	Amu Darya	Sherobod river	37.747222	66.996666	25/07/2019
436.	Dzihunia_sp3_Chirchik_626	SWU18082019626	FFU645-21	MW649562	Nemacheilidae	*Dzihunia* sp3	Sheraliev, BM	Sheraliev, BM	Tashkent	Syr Darya	Chirchik river	41.450555	69.598611	18/08/2019
437.	Dzihunia_sp3_Chirchik_627	SWU18082019627	FFU646-21	MW649563	Nemacheilidae	*Dzihunia* sp3	Sheraliev, BM	Sheraliev, BM	Tashkent	Syr Darya	Chirchik river	41.450555	69.598611	18/08/2019
438.	Dzihunia_sp3_Chirchik_628	SWU18082019628	FFU647-21	MW649564	Nemacheilidae	*Dzihunia* sp3	Sheraliev, BM	Sheraliev, BM	Tashkent	Syr Darya	Chirchik river	41.450555	69.598611	18/08/2019
439.	Dzihunia_sp3_Chirchik_629	SWU18082019629	FFU648-21	MW649565	Nemacheilidae	*Dzihunia* sp3	Sheraliev, BM	Sheraliev, BM	Tashkent	Syr Darya	Chirchik river	41.450555	69.598611	18/08/2019
440.	Dzihunia_sp3_Chirchik_635	SWU18082019635	FFU649-21	MW649566	Nemacheilidae	*Dzihunia* sp3	Sheraliev, BM	Sheraliev, BM	Tashkent	Syr Darya	Chirchik river	41.450555	69.598611	18/08/2019
441.	Dzihunia_sp3_Chirchik_636	SWU18082019636	FFU650-21	MW649567	Nemacheilidae	*Dzihunia* sp3	Sheraliev, BM	Sheraliev, BM	Tashkent	Syr Darya	Chirchik river	41.450555	69.598611	18/08/2019

442.	Dzihunia_sp3_Chirchik_637	SWU18082019637	FFU651-21	MW649568	Nemacheilidae	*Dzihunia* sp3	Sheraliev, BM	Sheraliev, BM	Tashkent	Syr Darya	Chirchik river	41.450555	69.598611	18/08/2019
443.	Dzihunia_sp3_Chirchik_638	SWU18082019638	FFU652-21	MW649569	Nemacheilidae	*Dzihunia* sp3	Sheraliev, BM	Sheraliev, BM	Tashkent	Syr Darya	Chirchik river	41.450555	69.598611	18/08/2019
444.	Paracobitis_longicauda_004	SWU08082016004	FFU417-21	MW649570	Nemacheilidae	*Paracobitis longicauda*	Sheraliev, BM	Sheraliev, BM	Surxondaryo	Amu Darya	Tupalang river	38.399802	67.944766	08/08/2016
445.	Paracobitis_longicauda_005	SWU08082016005	FFU418-21	MW649571	Nemacheilidae	*Paracobitis longicauda*	Sheraliev, BM	Sheraliev, BM	Surxondaryo	Amu Darya	Tupalang river	38.399802	67.944766	08/08/2016
446.	Paracobitis_longicauda_083	SWU08082016083	FFU419-21	MW649572	Nemacheilidae	*Paracobitis longicauda*	Sheraliev, BM	Sheraliev, BM	Surxondaryo	Amu Darya	Tupalang river	38.399802	67.944766	08/08/2016
447.	Paracobitis_longicauda_242	SWU08082016242	FFU420-21	MW649573	Nemacheilidae	*Paracobitis longicauda*	Sheraliev, BM	Sheraliev, BM	Surxondaryo	Amu Darya	Tupalang river	38.399802	67.944766	08/08/2016
448.	Paracobitis_longicauda_325	SWU21072019325	FFU421-21	MW649574	Nemacheilidae	*Paracobitis longicauda*	Sheraliev, BM	Sheraliev, BM	Surxondaryo	Amu Darya	Surkhan Darya	37.238888	67.329722	21/07/2019
449.	Paracobitis_longicauda_344	SWU22072019344	FFU422-21	MW649575	Nemacheilidae	*Paracobitis longicauda*	Sheraliev, BM	Sheraliev, BM	Surxondaryo	Amu Darya	Karatag river	38.363055	68.072059	22/07/2019
450.	Paracobitis_longicauda_345	SWU22072019345	FFU423-21	MW649576	Nemacheilidae	*Paracobitis longicauda*	Sheraliev, BM	Sheraliev, BM	Surxondaryo	Amu Darya	Karatag river	38.363055	68.072059	22/07/2019
451.	Paracobitis_longicauda_346	SWU22072019346	FFU424-21	MW649577	Nemacheilidae	*Paracobitis longicauda*	Sheraliev, BM	Sheraliev, BM	Surxondaryo	Amu Darya	Karatag river	38.363055	68.072059	22/07/2019
452.	Paracobitis_longicauda_347	SWU22072019347	FFU425-21	MW649578	Nemacheilidae	*Paracobitis longicauda*	Sheraliev, BM	Sheraliev, BM	Surxondaryo	Amu Darya	Karatag river	38.363055	68.072059	22/07/2019
453.	Paracobitis_longicauda_348	SWU22072019348	FFU426-21	MW649579	Nemacheilidae	*Paracobitis longicauda*	Sheraliev, BM	Sheraliev, BM	Surxondaryo	Amu Darya	Karatag river	38.363055	68.072059	22/07/2019
454.	Paracobitis_longicauda_349	SWU22072019349	FFU427-21	MW649580	Nemacheilidae	*Paracobitis longicauda*	Sheraliev, BM	Sheraliev, BM	Surxondaryo	Amu Darya	Karatag river	38.363055	68.072059	22/07/2019
455.	Paracobitis_longicauda_350	SWU22072019350	FFU428-21	MW649581	Nemacheilidae	*Paracobitis longicauda*	Sheraliev, BM	Sheraliev, BM	Surxondaryo	Amu Darya	Karatag river	38.363055	68.072059	22/07/2019
456.	Paracobitis_longicauda_351	SWU22072019351	FFU429-21	MW649582	Nemacheilidae	*Paracobitis longicauda*	Sheraliev, BM	Sheraliev, BM	Surxondaryo	Amu Darya	Karatag river	38.363055	68.072059	22/07/2019
457.	Paracobitis_longicauda_352	SWU22072019352	FFU430-21	MW649583	Nemacheilidae	*Paracobitis longicauda*	Sheraliev, BM	Sheraliev, BM	Surxondaryo	Amu Darya	Karatag river	38.363055	68.072059	22/07/2019
458.	Paracobitis_longicauda_375	SWU23072019375	FFU431-21	MW649584	Nemacheilidae	*Paracobitis longicauda*	Sheraliev, BM	Sheraliev, BM	Surxondaryo	Amu Darya	Tupalang River	38.318966	68.006169	23/07/2019
459.	Paracobitis_longicauda_407	SWU25072019407	FFU432-21	MW649585	Nemacheilidae	*Paracobitis longicauda*	Sheraliev, BM	Sheraliev, BM	Surxondaryo	Amu Darya	Sherobod river	37.747222	66.996666	25/07/2019
460.	Paracobitis_longicauda_408	SWU25072019408	FFU433-21	MW649586	Nemacheilidae	*Paracobitis longicauda*	Sheraliev, BM	Sheraliev, BM	Surxondaryo	Amu Darya	Sherobod river	37.747222	66.996666	25/07/2019
461.	Paracobitis_longicauda_502	SWU02082019502	FFU434-21	MW649587	Nemacheilidae	*Paracobitis longicauda*	Sheraliev, BM	Sheraliev, BM	Samarkand	Zeravshan	Zeravshan	39.676388	67.076111	02/08/2019
462.	Paracobitis_longicauda_683	SWU27042019683	FFU435-21	MW649588	Nemacheilidae	*Paracobitis longicauda*	Allayarov, S	Sheraliev, BM	Surxondaryo	Amu Darya	Karatag river	38.363055	68.072059	27/04/2019
463.	Paracobitis_longicauda_684	SWU27042019684	FFU436-21	MW649589	Nemacheilidae	*Paracobitis longicauda*	Allayarov, S	Sheraliev, BM	Surxondaryo	Amu Darya	Karatag river	38.363055	68.072059	27/04/2019
464.	Paracobitis_longicauda_685	SWU27042019685	FFU437-21	MW649590	Nemacheilidae	*Paracobitis longicauda*	Allayarov, S	Sheraliev, BM	Surxondaryo	Amu Darya	Karatag river	38.363055	68.072059	27/04/2019
465.	Paracobitis_longicauda_686	SWU27042019686	FFU438-21	MW649591	Nemacheilidae	*Paracobitis longicauda*	Allayarov, S	Sheraliev, BM	Surxondaryo	Amu Darya	Karatag river	38.363055	68.072059	27/04/2019
466.	Paracobitis_longicauda_687	SWU27042019687	FFU439-21	MW649592	Nemacheilidae	*Paracobitis longicauda*	Allayarov, S	Sheraliev, BM	Surxondaryo	Amu Darya	Karatag river	38.363055	68.072059	27/04/2019
467.	Paracobitis_longicauda_688	SWU27042019688	FFU440-21	MW649593	Nemacheilidae	*Paracobitis longicauda*	Allayarov, S	Sheraliev, BM	Surxondaryo	Amu Darya	Karatag river	38.363055	68.072059	27/04/2019
468.	Paracobitis_longicauda_689	SWU27042019689	FFU441-21	MW649594	Nemacheilidae	*Paracobitis longicauda*	Allayarov, S	Sheraliev, BM	Surxondaryo	Amu Darya	Karatag river	38.363055	68.072059	27/04/2019
469.	Triplophysa_sp1_Zeravshan_503	SWU02082019503	FFU604-21	MW649595	Nemacheilidae	*Triplophysa* sp.1	Sheraliev, BM	Sheraliev, BM	Samarkand	Zeravshan	Zeravshan	39.676388	67.076111	02/08/2019
470.	Triplophysa_sp1_Zeravshan_513	SWU02082019513	FFU605-21	MW649596	Nemacheilidae	*Triplophysa* sp.1	Sheraliev, BM	Sheraliev, BM	Samarkand	Zeravshan	Zeravshan	39.676388	67.076111	02/08/2019
471.	Triplophysa_sp1_Zeravshan_514	SWU02082019514	FFU606-21	MW649597	Nemacheilidae	*Triplophysa* sp.1	Sheraliev, BM	Sheraliev, BM	Samarkand	Zeravshan	Zeravshan	39.676388	67.076111	02/08/2019
472.	Triplophysa_sp1_Zeravshan_515	SWU02082019515	FFU607-21	MW649598	Nemacheilidae	*Triplophysa* sp.1	Sheraliev, BM	Sheraliev, BM	Samarkand	Zeravshan	Zeravshan	39.676388	67.076111	02/08/2019
473.	Triplophysa_sp2_Chirchik_630	SWU18082019630	FFU608-21	MW649599	Nemacheilidae	*Triplophysa* sp.2	Sheraliev, BM	Sheraliev, BM	Tashkent	Syr Darya	Chirchik river	41.450555	69.598611	18/08/2019
474.	Triplophysa_sp2_Chirchik_631	SWU18082019631	FFU609-21	MW649600	Nemacheilidae	*Triplophysa* sp.2	Sheraliev, BM	Sheraliev, BM	Tashkent	Syr Darya	Chirchik river	41.450555	69.598611	18/08/2019
475.	Triplophysa_sp2_Chirchik_632	SWU18082019632	FFU610-21	MW649601	Nemacheilidae	*Triplophysa* sp.2	Sheraliev, BM	Sheraliev, BM	Tashkent	Syr Darya	Chirchik river	41.450555	69.598611	18/08/2019
476.	Triplophysa_sp2_Chirchik_633	SWU18082019633	FFU611-21	MW649602	Nemacheilidae	*Triplophysa* sp.2	Sheraliev, BM	Sheraliev, BM	Tashkent	Syr Darya	Chirchik river	41.450555	69.598611	18/08/2019
477.	Triplophysa_sp3_Fergana_647	SWU13082019647	FFU612-21	MW649603	Nemacheilidae	*Triplophysa ferganaensis*	Sheraliev, BM	Sheraliev, BM	Fergana	Syr Darya	Shohimardon River	39.963088	71.759857	13/08/2019
478.	Triplophysa_sp3_Fergana_648	SWU13082019648	FFU613-21	MW649604	Nemacheilidae	*Triplophysa ferganaensis*	Sheraliev, BM	Sheraliev, BM	Fergana	Syr Darya	Shohimardon River	39.963088	71.759857	13/08/2019
479.	Triplophysa_sp3_Fergana_649	SWU13082019649	FFU614-21	MW649605	Nemacheilidae	*Triplophysa ferganaensis*	Sheraliev, BM	Sheraliev, BM	Fergana	Syr Darya	Shohimardon River	39.963088	71.759857	13/08/2019
480.	Triplophysa_sp3_Fergana_650	SWU13082019650	FFU615-21	MW649606	Nemacheilidae	*Triplophysa ferganaensis*	Sheraliev, BM	Sheraliev, BM	Fergana	Syr Darya	Shohimardon River	39.963088	71.759857	13/08/2019
481.	Triplophysa_sp3_Fergana_651	SWU13082019651	FFU616-21	MW649607	Nemacheilidae	*Triplophysa ferganaensis*	Sheraliev, BM	Sheraliev, BM	Fergana	Syr Darya	Shohimardon River	39.963088	71.759857	13/08/2019
482.	Triplophysa_sp3_Fergana_652	SWU13082019652	FFU617-21	MW649608	Nemacheilidae	*Triplophysa ferganaensis*	Sheraliev, BM	Sheraliev, BM	Fergana	Syr Darya	Shohimardon River	39.963088	71.759857	13/08/2019
483.	Triplophysa_sp3_Fergana_653	SWU13082019653	FFU618-21	MW649609	Nemacheilidae	*Triplophysa ferganaensis*	Sheraliev, BM	Sheraliev, BM	Fergana	Syr Darya	Shohimardon River	39.963088	71.759857	13/08/2019
484.	Triplophysa_sp3_Fergana_654	SWU13082019654	FFU619-21	MW649610	Nemacheilidae	*Triplophysa ferganaensis*	Sheraliev, BM	Sheraliev, BM	Fergana	Syr Darya	Shohimardon River	39.963088	71.759857	13/08/2019
485.	Triplophysa_sp3_Fergana_655	SWU13082019655	FFU620-21	MW649611	Nemacheilidae	*Triplophysa ferganaensis*	Sheraliev, BM	Sheraliev, BM	Fergana	Syr Darya	Shohimardon River	39.963088	71.759857	13/08/2019
486.	Triplophysa_sp3_Fergana_656	SWU13082019656	FFU621-21	MW649612	Nemacheilidae	*Triplophysa ferganaensis*	Sheraliev, BM	Sheraliev, BM	Fergana	Syr Darya	Shohimardon River	39.963088	71.759857	13/08/2019
487.	Triplophysa_sp3_Fergana_657	SWU13082019657	FFU622-21	MW649613	Nemacheilidae	*Triplophysa ferganaensis*	Sheraliev, BM	Sheraliev, BM	Fergana	Syr Darya	Shohimardon River	39.963088	71.759857	13/08/2019
488.	Triplophysa_sp3_Fergana_658	SWU13082019658	FFU623-21	MW649614	Nemacheilidae	*Triplophysa ferganaensis*	Sheraliev, BM	Sheraliev, BM	Fergana	Syr Darya	Shohimardon River	39.963088	71.759857	13/08/2019
489.	Triplophysa_sp3_Fergana_659	SWU13082019659	FFU624-21	MW649615	Nemacheilidae	*Triplophysa ferganaensis*	Sheraliev, BM	Sheraliev, BM	Fergana	Syr Darya	Shohimardon River	39.963088	71.759857	13/08/2019
490.	Triplophysa_sp3_Fergana_660	SWU13082019660	FFU625-21	MW649616	Nemacheilidae	*Triplophysa ferganaensis*	Sheraliev, BM	Sheraliev, BM	Fergana	Syr Darya	Shohimardon River	39.963088	71.759857	13/08/2019
491.	Triplophysa_sp3_Fergana_661	SWU13082019661	FFU626-21	MW649617	Nemacheilidae	*Triplophysa ferganaensis*	Sheraliev, BM	Sheraliev, BM	Fergana	Syr Darya	Shohimardon River	39.963088	71.759857	13/08/2019
492.	Triplophysa_sp3_Fergana_662	SWU13082019662	FFU627-21	MW649618	Nemacheilidae	*Triplophysa ferganaensis*	Sheraliev, BM	Sheraliev, BM	Fergana	Syr Darya	Shohimardon River	39.963088	71.759857	13/08/2019
493.	Triplophysa_sp3_Fergana_663	SWU13082019663	FFU628-21	MW649619	Nemacheilidae	*Triplophysa ferganaensis*	Sheraliev, BM	Sheraliev, BM	Fergana	Syr Darya	Shohimardon River	39.963088	71.759857	13/08/2019
494.	Triplophysa_sp3_Fergana_664	SWU13082019664	FFU629-21	MW649620	Nemacheilidae	*Triplophysa ferganaensis*	Sheraliev, BM	Sheraliev, BM	Fergana	Syr Darya	Shohimardon River	39.963088	71.759857	13/08/2019
495.	Triplophysa_sp3_Fergana_665	SWU13082019665	FFU630-21	MW649621	Nemacheilidae	*Triplophysa ferganaensis*	Sheraliev, BM	Sheraliev, BM	Fergana	Syr Darya	Shohimardon River	39.963088	71.759857	13/08/2019
496.	Triplophysa_sp3_Fergana_666	SWU13082019666	FFU631-21	MW649622	Nemacheilidae	*Triplophysa ferganaensis*	Sheraliev, BM	Sheraliev, BM	Fergana	Syr Darya	Shohimardon River	39.963088	71.759857	13/08/2019
497.	Triplophysa_strauchii_052	SWU07012017052	FFU442-21	MW649623	Nemacheilidae	*Triplophysa strauchii*	Sheraliev, BM	Sheraliev, BM	Fergana	Syr Darya	Unnamed stream	40.366437	71.316907	07/01/2017
498.	Triplophysa_strauchii_053	SWU07012017053	FFU443-21	MW649624	Nemacheilidae	*Triplophysa strauchii*	Sheraliev, BM	Sheraliev, BM	Fergana	Syr Darya	Unnamed stream	40.366437	71.316907	07/01/2017
499.	Triplophysa_strauchii_056	SWU07012017056	FFU444-21	MW649625	Nemacheilidae	*Triplophysa strauchii*	Sheraliev, BM	Sheraliev, BM	Fergana	Syr Darya	Unnamed stream	40.366437	71.316907	07/01/2017
500.	Triplophysa_strauchii_104	SWU25012018104	FFU445-21	MW649626	Nemacheilidae	*Triplophysa strauchii*	Sheraliev, BM	Sheraliev, BM	Fergana	Syr Darya	Fergana Canal	40.388497	71.327960	25/01/2018
501.	Triplophysa_strauchii_105	SWU25012018105	FFU446-21	MW649627	Nemacheilidae	*Triplophysa strauchii*	Sheraliev, BM	Sheraliev, BM	Fergana	Syr Darya	Fergana Canal	40.388497	71.327960	25/01/2018
502.	Triplophysa_strauchii_106	SWU25012018106	FFU447-21	MW649628	Nemacheilidae	*Triplophysa strauchii*	Sheraliev, BM	Sheraliev, BM	Fergana	Syr Darya	Fergana Canal	40.388497	71.327960	25/01/2018
503.	Triplophysa_strauchii_129	SWU21022018129	FFU448-21	MW649629	Nemacheilidae	*Triplophysa strauchii*	Sheraliev, BM	Sheraliev, BM	Tashkent	Syr Darya	Chirchik River	41.370266	69.512026	21/02/2018
504.	Triplophysa_strauchii_130	SWU21022018130	FFU449-21	MW649630	Nemacheilidae	*Triplophysa strauchii*	Sheraliev, BM	Sheraliev, BM	Tashkent	Syr Darya	Chirchik River	41.370266	69.512026	21/02/2018
505.	Triplophysa_strauchii_204	SWU11082018204	FFU450-21	MW649631	Nemacheilidae	*Triplophysa strauchii*	Sheraliev, BM	Sheraliev, BM	Fergana	Syr Darya	Unnamed stream	40.312769	71.811647	11/08/2018
506.	Triplophysa_strauchii_205	SWU11082018205	FFU451-21	MW649632	Nemacheilidae	*Triplophysa strauchii*	Sheraliev, BM	Sheraliev, BM	Fergana	Syr Darya	Unnamed stream	40.312769	71.811647	11/08/2018
507.	Triplophysa_strauchii_206	SWU11082018206	FFU452-21	MW649633	Nemacheilidae	*Triplophysa strauchii*	Sheraliev, BM	Sheraliev, BM	Fergana	Syr Darya	Unnamed stream	40.312769	71.811647	11/08/2018
508.	Triplophysa_strauchii_207	SWU11082018207	FFU453-21	MW649634	Nemacheilidae	*Triplophysa strauchii*	Sheraliev, BM	Sheraliev, BM	Fergana	Syr Darya	Unnamed stream	40.312769	71.811647	11/08/2018
509.	Triplophysa_strauchii_208	SWU11082018208	FFU454-21	MW649635	Nemacheilidae	*Triplophysa strauchii*	Sheraliev, BM	Sheraliev, BM	Fergana	Syr Darya	Unnamed stream	40.312769	71.811647	11/08/2018
510.	Triplophysa_strauchii_210	SWU11082018210	FFU455-21	MW649636	Nemacheilidae	*Triplophysa strauchii*	Sheraliev, BM	Sheraliev, BM	Fergana	Syr Darya	Unnamed stream	40.312769	71.811647	11/08/2018
511.	Triplophysa_strauchii_230	SWU15012017230	FFU456-21	MW649637	Nemacheilidae	*Triplophysa strauchii*	Sheraliev, BM	Sheraliev, BM	Fergana	Syr Darya	Unnamed stream	40.326408	71.820508	15/01/2017
512.	Triplophysa_strauchii_231	SWU15012017231	FFU457-21	MW649638	Nemacheilidae	*Triplophysa strauchii*	Sheraliev, BM	Sheraliev, BM	Fergana	Syr Darya	Unnamed stream	40.326408	71.820508	15/01/2017
513.	Triplophysa_strauchii_248	SWU11082018248	FFU458-21	MW649639	Nemacheilidae	*Triplophysa strauchii*	Sheraliev, BM	Sheraliev, BM	Fergana	Syr Darya	Unnamed stream	40.312769	71.811647	11/08/2018
514.	Triplophysa_strauchii_249	SWU11082018249	FFU459-21	MW649640	Nemacheilidae	*Triplophysa strauchii*	Sheraliev, BM	Sheraliev, BM	Fergana	Syr Darya	Unnamed stream	40.312769	71.811647	11/08/2018
515.	Triplophysa_strauchii_551	SWU09082019551	FFU460-21	MW649641	Nemacheilidae	*Triplophysa strauchii*	Sheraliev, BM	Sheraliev, BM	Namangan	Syr Darya	Kara Darya	40.918813	71.828896	09/08/2019
516.	Triplophysa_strauchii_617	SWU18082019617	FFU461-21	MW649642	Nemacheilidae	*Triplophysa strauchii*	Sheraliev, BM	Sheraliev, BM	Tashkent	Syr Darya	Chirchik River	41.274444	69.389166	18/08/2019
517.	Triplophysa_strauchii_618	SWU18082019618	FFU462-21	MW649643	Nemacheilidae	*Triplophysa strauchii*	Sheraliev, BM	Sheraliev, BM	Tashkent	Syr Darya	Chirchik River	41.274444	69.389166	18/08/2019
518.	Triplophysa_strauchii_619	SWU18082019619	FFU463-21	MW649644	Nemacheilidae	*Triplophysa strauchii*	Sheraliev, BM	Sheraliev, BM	Tashkent	Syr Darya	Chirchik River	41.274444	69.389166	18/08/2019

519.	Triplophysa_strauchii_621	SWU18082019621	FFU464-21	MW649645	Nemacheilidae	*Triplophysa strauchii*	Sheraliev, BM	Sheraliev, BM	Tashkent	Syr Darya	Chirchik River	41.274444	69.389166	18/08/2019
520.	Triplophysa_strauchii_622	SWU18082019622	FFU465-21	MW649646	Nemacheilidae	*Triplophysa strauchii*	Sheraliev, BM	Sheraliev, BM	Tashkent	Syr Darya	Chirchik River	41.274444	69.389166	18/08/2019
521.	Triplophysa_strauchii_623	SWU18082019623	FFU466-21	MW649647	Nemacheilidae	*Triplophysa strauchii*	Sheraliev, BM	Sheraliev, BM	Tashkent	Syr Darya	Chirchik River	41.274444	69.389166	18/08/2019
522.	Triplophysa_strauchii_624	SWU18082019624	FFU467-21	MW649648	Nemacheilidae	*Triplophysa strauchii*	Sheraliev, BM	Sheraliev, BM	Tashkent	Syr Darya	Chirchik River	41.274444	69.389166	18/08/2019
523.	Triplophysa_strauchii_625	SWU18082019625	FFU468-21	MW649649	Nemacheilidae	*Triplophysa strauchii*	Sheraliev, BM	Sheraliev, BM	Tashkent	Syr Darya	Chirchik River	41.274444	69.389166	18/08/2019
524.	Triplophysa_strauchii_642	SWU20082019642	FFU469-21	MW649650	Nemacheilidae	*Triplophysa strauchii*	Sheraliev, BM	Sheraliev, BM	Fergana	Syr Darya	Unnamed stream	40.305260	71.800688	20/08/2019
525.	Triplophysa_strauchii_643	SWU20082019643	FFU470-21	MW649651	Nemacheilidae	*Triplophysa strauchii*	Sheraliev, BM	Sheraliev, BM	Fergana	Syr Darya	Unnamed stream	40.305260	71.800688	20/08/2019
526.	Sander_lucioperca_009	SWU14082016009	FFU471-21	MW649652	Percidae	*Sander lucioperca*	Sheraliev, BM	Sheraliev, BM	Surxondaryo	Amu Darya	South Surkhan R.	37.854013	67.628373	14/08/2016
527.	Sander_lucioperca_010	SWU14082016010	FFU472-21	MW649653	Percidae	*Sander lucioperca*	Sheraliev, BM	Sheraliev, BM	Surxondaryo	Amu Darya	South Surkhan R.	37.854013	67.628373	14/08/2016
528.	Sander_lucioperca_185	SWU18072018185	FFU473-21	MW649654	Percidae	*Sander lucioperca*	Sheraliev, BM	Sheraliev, BM	Navoiy	Zeravshan	Tudakul Lake	39.801388	64.772222	18/07/2018
529.	Sander_lucioperca_186	SWU18072018186	FFU474-21	MW649655	Percidae	*Sander lucioperca*	Sheraliev, BM	Sheraliev, BM	Navoiy	Zeravshan	Tudakul Lake	39.801388	64.772222	18/07/2018
530.	Sander_lucioperca_187	SWU18072018187	FFU475-21	MW649656	Percidae	*Sander lucioperca*	Sheraliev, BM	Sheraliev, BM	Navoiy	Zeravshan	Tudakul Lake	39.801388	64.772222	18/07/2018
531.	Sander_lucioperca_188	SWU18072018188	FFU476-21	MW649657	Percidae	*Sander lucioperca*	Sheraliev, BM	Sheraliev, BM	Navoiy	Zeravshan	Tudakul Lake	39.801388	64.772222	18/07/2018
532.	Sander_lucioperca_189	SWU18072018189	FFU477-21	MW649658	Percidae	*Sander lucioperca*	Sheraliev, BM	Sheraliev, BM	Navoiy	Zeravshan	Tudakul Lake	39.801388	64.772222	18/07/2018
533.	Sander_lucioperca_439	SWU27072019439	FFU478-21	MW649659	Percidae	*Sander lucioperca*	Sheraliev, BM	Sheraliev, BM	Bukhara	Zeravshan	Echkiliksoy	40.267551	64.743333	27/07/2019
534.	Sander_lucioperca_446	SWU28072019446	FFU479-21	MW649660	Percidae	*Sander lucioperca*	Sheraliev, BM	Sheraliev, BM	Bukhara	Zeravshan	Zeravshan	40.071012	64.778429	28/07/2019
535.	Sander_lucioperca_477	SWU30072019477	FFU480-21	MW649661	Percidae	*Sander lucioperca*	Sheraliev, BM	Sheraliev, BM	Khorazm	Amu Darya	Unnamed canal	41.282666	60.803916	30/07/2019
536.	Gambusia_holbrooki_140	SWU25012018140	FFU481-21	MW649662	Poeciliidae	*Gambusia holbrooki*	Sheraliev, BM	Sheraliev, BM	Fergana	Syr Darya	Fergana Canal	40.396753	71.407328	25/01/2018
537.	Gambusia_holbrooki_141	SWU25012018141	FFU482-21	MW649663	Poeciliidae	*Gambusia holbrooki*	Sheraliev, BM	Sheraliev, BM	Fergana	Syr Darya	Fergana Canal	40.396753	71.407328	25/01/2018
538.	Gambusia_holbrooki_142	SWU25012018142	FFU483-21	MW649664	Poeciliidae	*Gambusia holbrooki*	Sheraliev, BM	Sheraliev, BM	Fergana	Syr Darya	Fergana Canal	40.396753	71.407328	25/01/2018
539.	Gambusia_holbrooki_143	SWU25012018143	FFU484-21	MW649665	Poeciliidae	*Gambusia holbrooki*	Sheraliev, BM	Sheraliev, BM	Fergana	Syr Darya	Fergana Canal	40.396753	71.407328	25/01/2018
540.	Gambusia_holbrooki_216	SWU16072018216	FFU485-21	MW649666	Poeciliidae	*Gambusia holbrooki*	Sheraliev, BM	Sheraliev, BM	Bukhara	Zeravshan	Zeravshan	40.146944	64.891666	16/07/2018
541.	Gambusia_holbrooki_217	SWU16072018217	FFU486-21	MW649667	Poeciliidae	*Gambusia holbrooki*	Sheraliev, BM	Sheraliev, BM	Bukhara	Zeravshan	Zeravshan	40.146944	64.891666	16/07/2018
542.	Gambusia_holbrooki_262	SWU19072019262	FFU487-21	MW649668	Poeciliidae	*Gambusia holbrooki*	Sheraliev, BM	Sheraliev, BM	Surxondaryo	Amu Darya	Surkhan Darya	37.255833	67.338611	19/07/2019
543.	Gambusia_holbrooki_263	SWU19072019263	FFU488-21	MW649669	Poeciliidae	*Gambusia holbrooki*	Sheraliev, BM	Sheraliev, BM	Surxondaryo	Amu Darya	Surkhan Darya	37.255833	67.338611	19/07/2019
544.	Gambusia_holbrooki_296	SWU21072019296	FFU489-21	MW649670	Poeciliidae	*Gambusia holbrooki*	Sheraliev, BM	Sheraliev, BM	Surxondaryo	Amu Darya	Surkhan Darya	37.238888	67.329722	21/07/2019
545.	Gambusia_holbrooki_297	SWU21072019297	FFU490-21	MW649671	Poeciliidae	*Gambusia holbrooki*	Sheraliev, BM	Sheraliev, BM	Surxondaryo	Amu Darya	Surkhan Darya	37.238888	67.329722	21/07/2019
546.	Gambusia_holbrooki_298	SWU21072019298	FFU491-21	MW649672	Poeciliidae	*Gambusia holbrooki*	Sheraliev, BM	Sheraliev, BM	Surxondaryo	Amu Darya	Surkhan Darya	37.238888	67.329722	21/07/2019
547.	Gambusia_holbrooki_299	SWU21072019299	FFU492-21	MW649673	Poeciliidae	*Gambusia holbrooki*	Sheraliev, BM	Sheraliev, BM	Surxondaryo	Amu Darya	Surkhan Darya	37.238888	67.329722	21/07/2019
548.	Gambusia_holbrooki_334	SWU22072019334	FFU493-21	MW649674	Poeciliidae	*Gambusia holbrooki*	Sheraliev, BM	Sheraliev, BM	Surxondaryo	Amu Darya	Karatag river	38.363055	68.072059	22/07/2019
549.	Gambusia_holbrooki_335	SWU22072019335	FFU494-21	MW649675	Poeciliidae	*Gambusia holbrooki*	Sheraliev, BM	Sheraliev, BM	Surxondaryo	Amu Darya	Karatag river	38.363055	68.072059	22/07/2019
550.	Gambusia_holbrooki_336	SWU22072019336	FFU495-21	MW649676	Poeciliidae	*Gambusia holbrooki*	Sheraliev, BM	Sheraliev, BM	Surxondaryo	Amu Darya	Karatag river	38.363055	68.072059	22/07/2019
551.	Gambusia_holbrooki_337	SWU22072019337	FFU496-21	MW649677	Poeciliidae	*Gambusia holbrooki*	Sheraliev, BM	Sheraliev, BM	Surxondaryo	Amu Darya	Karatag river	38.363055	68.072059	22/07/2019
552.	Gambusia_holbrooki_393	SWU24072019393	FFU497-21	MW649678	Poeciliidae	*Gambusia holbrooki*	Sheraliev, BM	Sheraliev, BM	Surxondaryo	Amu Darya	Surkhan Darya	37.823333	67.618611	24/07/2019
553.	Gambusia_holbrooki_394	SWU24072019394	FFU498-21	MW649679	Poeciliidae	*Gambusia holbrooki*	Sheraliev, BM	Sheraliev, BM	Surxondaryo	Amu Darya	Surkhan Darya	37.823333	67.618611	24/07/2019
554.	Gambusia_holbrooki_395	SWU24072019395	FFU499-21	MW649680	Poeciliidae	*Gambusia holbrooki*	Sheraliev, BM	Sheraliev, BM	Surxondaryo	Amu Darya	Surkhan Darya	37.823333	67.618611	24/07/2019
555.	Gambusia_holbrooki_396	SWU24072019396	FFU500-21	MW649681	Poeciliidae	*Gambusia holbrooki*	Sheraliev, BM	Sheraliev, BM	Surxondaryo	Amu Darya	Surkhan Darya	37.823333	67.618611	24/07/2019
556.	Gambusia_holbrooki_421	SWU27072019421	FFU501-21	MW649682	Poeciliidae	*Gambusia holbrooki*	Sheraliev, BM	Sheraliev, BM	Bukhara	Zeravshan	Echkiliksoy	40.267551	64.743333	27/07/2019
557.	Gambusia_holbrooki_422	SWU27072019422	FFU502-21	MW649683	Poeciliidae	*Gambusia holbrooki*	Sheraliev, BM	Sheraliev, BM	Bukhara	Zeravshan	Echkiliksoy	40.267551	64.743333	27/07/2019
558.	Gambusia_holbrooki_423	SWU27072019423	FFU503-21	MW649684	Poeciliidae	*Gambusia holbrooki*	Sheraliev, BM	Sheraliev, BM	Bukhara	Zeravshan	Echkiliksoy	40.267551	64.743333	27/07/2019
559.	Gambusia_holbrooki_424	SWU27072019424	FFU504-21	MW649685	Poeciliidae	*Gambusia holbrooki*	Sheraliev, BM	Sheraliev, BM	Bukhara	Zeravshan	Echkiliksoy	40.267551	64.743333	27/07/2019
560.	Gambusia_holbrooki_425	SWU27072019425	FFU505-21	MW649686	Poeciliidae	*Gambusia holbrooki*	Sheraliev, BM	Sheraliev, BM	Bukhara	Zeravshan	Echkiliksoy	40.267551	64.743333	27/07/2019
561.	Gambusia_holbrooki_463	SWU28072019463	FFU506-21	MW649687	Poeciliidae	*Gambusia holbrooki*	Sheraliev, BM	Sheraliev, BM	Bukhara	Zeravshan	Zeravshan	40.071012	64.778429	28/07/2019
562.	Gambusia_holbrooki_464	SWU28072019464	FFU507-21	MW649688	Poeciliidae	*Gambusia holbrooki*	Sheraliev, BM	Sheraliev, BM	Bukhara	Zeravshan	Zeravshan	40.071012	64.778429	28/07/2019
563.	Gambusia_holbrooki_525	SWU02082019525	FFU508-21	MW649689	Poeciliidae	*Gambusia holbrooki*	Sheraliev, BM	Sheraliev, BM	Samarkand	Zeravshan	Zeravshan	39.676388	67.076111	02/08/2019
564.	Gambusia_holbrooki_526	SWU02082019526	FFU509-21	MW649690	Poeciliidae	*Gambusia holbrooki*	Sheraliev, BM	Sheraliev, BM	Samarkand	Zeravshan	Zeravshan	39.676388	67.076111	02/08/2019
565.	Gambusia_holbrooki_527	SWU02082019527	FFU510-21	MW649691	Poeciliidae	*Gambusia holbrooki*	Sheraliev, BM	Sheraliev, BM	Samarkand	Zeravshan	Zeravshan	39.676388	67.076111	02/08/2019
566.	Gambusia_holbrooki_528	SWU02082019528	FFU511-21	MW649692	Poeciliidae	*Gambusia holbrooki*	Sheraliev, BM	Sheraliev, BM	Samarkand	Zeravshan	Zeravshan	39.676388	67.076111	02/08/2019
567.	Gambusia_holbrooki_529	SWU02082019529	FFU512-21	MW649693	Poeciliidae	*Gambusia holbrooki*	Sheraliev, BM	Sheraliev, BM	Samarkand	Zeravshan	Zeravshan	39.676388	67.076111	02/08/2019
568.	Gambusia_holbrooki_530	SWU02082019530	FFU513-21	MW649694	Poeciliidae	*Gambusia holbrooki*	Sheraliev, BM	Sheraliev, BM	Samarkand	Zeravshan	Zeravshan	39.676388	67.076111	02/08/2019
569.	Gambusia_holbrooki_559	SWU09082019559	FFU514-21	MW649695	Poeciliidae	*Gambusia holbrooki*	Sheraliev, BM	Sheraliev, BM	Namangan	Syr Darya	Kara Darya	40.918813	71.828896	09/08/2019
570.	Gambusia_holbrooki_560	SWU09082019560	FFU515-21	MW649696	Poeciliidae	*Gambusia holbrooki*	Sheraliev, BM	Sheraliev, BM	Namangan	Syr Darya	Kara Darya	40.918813	71.828896	09/08/2019
571.	Gambusia_holbrooki_561	SWU09082019561	FFU516-21	MW649697	Poeciliidae	*Gambusia holbrooki*	Sheraliev, BM	Sheraliev, BM	Namangan	Syr Darya	Kara Darya	40.918813	71.828896	09/08/2019
572.	Gambusia_holbrooki_562	SWU09082019562	FFU517-21	MW649698	Poeciliidae	*Gambusia holbrooki*	Sheraliev, BM	Sheraliev, BM	Namangan	Syr Darya	Kara Darya	40.918813	71.828896	09/08/2019
573.	Gambusia_holbrooki_598	SWU18082019598	FFU518-21	MW649699	Poeciliidae	*Gambusia holbrooki*	Sheraliev, BM	Sheraliev, BM	Tashkent	Syr Darya	Chirchik river	41.274444	69.389166	18/08/2019
574.	Gambusia_holbrooki_599	SWU18082019599	FFU519-21	MW649700	Poeciliidae	*Gambusia holbrooki*	Sheraliev, BM	Sheraliev, BM	Tashkent	Syr Darya	Chirchik river	41.274444	69.389166	18/08/2019
575.	Gambusia_holbrooki_600	SWU18082019600	FFU520-21	MW649701	Poeciliidae	*Gambusia holbrooki*	Sheraliev, BM	Sheraliev, BM	Tashkent	Syr Darya	Chirchik river	41.274444	69.389166	18/08/2019
576.	Gambusia_holbrooki_601	SWU18082019601	FFU521-21	MW649702	Poeciliidae	*Gambusia holbrooki*	Sheraliev, BM	Sheraliev, BM	Tashkent	Syr Darya	Chirchik river	41.274444	69.389166	18/08/2019
577.	Gambusia_holbrooki_675	SWU21082019675	FFU522-21	MW649703	Poeciliidae	*Gambusia holbrooki*	Rozimov, A	Sheraliev, BM	Khorazm	Amu Darya	Unnamed canal	41.521638	60.566111	21/08/2019
578.	Gambusia_holbrooki_676	SWU21082019676	FFU523-21	MW649704	Poeciliidae	*Gambusia holbrooki*	Rozimov, A	Sheraliev, BM	Khorazm	Amu Darya	Unnamed canal	41.521638	60.566111	21/08/2019
579.	Gambusia_holbrooki_677	SWU21082019677	FFU524-21	MW649705	Poeciliidae	*Gambusia holbrooki*	Rozimov, A	Sheraliev, BM	Khorazm	Amu Darya	Unnamed canal	41.521638	60.566111	21/08/2019
580.	Oncorhynchus_mykiss_704	SWU03012020704	FFU525-21	MW649706	Salmonidae	*Oncorhynchus mykiss*	Xushboqov, Q	Sheraliev, BM	Surxondaryo	Amu Darya	Tupalang	38.570627	67.805326	03/01/2020
581.	Oncorhynchus_mykiss_705	SWU03012020705	FFU526-21	MW649707	Salmonidae	*Oncorhynchus mykiss*	Xushboqov, Q	Sheraliev, BM	Surxondaryo	Amu Darya	Tupalang	38.570627	67.805326	03/01/2020
582.	Silurus_glanis_050	SWU31082016050	FFU527-21	MW649708	Siluridae	*Silurus glanis*	Sheraliev, BM	Sheraliev, BM	Namangan	Syr Darya	Syr Darya	40.882777	71.442222	31/08/2016
583.	Silurus_glanis_051	SWU31082016051	FFU528-21	MW649709	Siluridae	*Silurus glanis*	Sheraliev, BM	Sheraliev, BM	Namangan	Syr Darya	Syr Darya	40.882777	71.442222	31/08/2016
584.	Silurus_glanis_176	SWU18072018176	FFU529-21	MW649710	Siluridae	*Silurus glanis*	Sheraliev, BM	Sheraliev, BM	Navoiy	Zeravshan	Tudakul Lake	39.801388	64.772222	18/07/2018
585.	Silurus_glanis_281	SWU20072019281	FFU530-21	MW649711	Siluridae	*Silurus glanis*	Sheraliev, BM	Sheraliev, BM	Surxondaryo	Amu Darya	Amu Darya	37.235555	67.660555	20/07/2019
586.	Silurus_glanis_282	SWU20072019282	FFU531-21	MW649712	Siluridae	*Silurus glanis*	Sheraliev, BM	Sheraliev, BM	Surxondaryo	Amu Darya	Amu Darya	37.235555	67.660555	20/07/2019
587.	Silurus_glanis_283	SWU20072019283	FFU532-21	MW649713	Siluridae	*Silurus glanis*	Sheraliev, BM	Sheraliev, BM	Surxondaryo	Amu Darya	Amu Darya	37.235555	67.660555	20/07/2019
588.	Silurus_glanis_667	SWU21082019667	FFU533-21	MW649714	Siluridae	*Silurus glanis*	Rozimov, A	Sheraliev, BM	Khorazm	Amu Darya	Amu Darya	41.880305	60.450888	21/08/2019
589.	Silurus_glanis_668	SWU21082019668	FFU534-21	MW649715	Siluridae	*Silurus glanis*	Rozimov, A	Sheraliev, BM	Khorazm	Amu Darya	Amu Darya	41.880305	60.450888	21/08/2019
590.	Glyptosternon_oschanini_576	SWU09082019576	FFU653-21	MW649716	Sisoridae	*Glyptosternon oschanini*	Sheraliev, BM	Sheraliev, BM	Namangan	Syr Darya	Kara Darya	40.918813	71.828896	09/08/2019
591.	Glyptosternon_sp_084	SWU08082016084	FFU654-21	MW649717	Sisoridae	*Glyptosternon* sp.	Sheraliev, BM	Sheraliev, BM	Surxondaryo	Amu Darya	Tupalang river	38.541702	67.823001	08/08/2016
592.	Glyptosternon_sp_085	SWU08082016085	FFU655-21	MW649718	Sisoridae	*Glyptosternon* sp.	Sheraliev, BM	Sheraliev, BM	Surxondaryo	Amu Darya	Tupalang river	38.541702	67.823001	08/08/2016
593.	Glyptosternon_sp_087	SWU08082016087	FFU656-21	MW649719	Sisoridae	*Glyptosternon* sp.	Sheraliev, BM	Sheraliev, BM	Surxondaryo	Amu Darya	Tupalang river	38.541702	67.823001	08/08/2016
594.	Glyptosternon_sp_088	SWU08082016088	FFU657-21	MW649720	Sisoridae	*Glyptosternon* sp.	Sheraliev, BM	Sheraliev, BM	Surxondaryo	Amu Darya	Tupalang river	38.541702	67.823001	08/08/2016
595.	Glyptosternon_sp_218	SWU08082016218	FFU658-21	MW649721	Sisoridae	*Glyptosternon* sp.	Sheraliev, BM	Sheraliev, BM	Surxondaryo	Amu Darya	Tupalang river	38.541702	67.823001	08/08/2016

596.	Glyptosternon_sp_220	SWU08082016220	FFU659-21	MW649722	Sisoridae	*Glyptosternon* sp.	Sheraliev, BM	Sheraliev, BM	Surxondaryo	Amu Darya	Tupalang river	38.541702	67.823001	08/08/2016
597.	Glyptosternon_sp_221	SWU08082016221	FFU660-21	MW649723	Sisoridae	*Glyptosternon* sp.	Sheraliev, BM	Sheraliev, BM	Surxondaryo	Amu Darya	Tupalang river	38.541702	67.823001	08/08/2016
598.	Glyptosternon_sp_222	SWU08082016222	FFU661-21	MW649724	Sisoridae	*Glyptosternon* sp.	Sheraliev, BM	Sheraliev, BM	Surxondaryo	Amu Darya	Tupalang river	38.541702	67.823001	08/08/2016
599.	Ctenopharyngodon_idella_107	SWU28012018107	FFU535-21	MW649725	Xenocyprididae	*Ctenopharyngodon idella*	Sheraliev, BM	Sheraliev, BM	Fergana	Syr Darya	Local fish market			28/01/2018
600.	Ctenopharyngodon_idella_108	SWU28012018108	FFU536-21	MW649726	Xenocyprididae	*Ctenopharyngodon idella*	Sheraliev, BM	Sheraliev, BM	Fergana	Syr Darya	Local fish market			28/01/2018
601.	Ctenopharyngodon_idella_109	SWU28012018109	FFU537-21	MW649727	Xenocyprididae	*Ctenopharyngodon idella*	Sheraliev, BM	Sheraliev, BM	Fergana	Syr Darya	Local fish market			28/01/2018
602.	Ctenopharyngodon_idella_110	SWU28012018110	FFU538-21	MW649728	Xenocyprididae	*Ctenopharyngodon idella*	Sheraliev, BM	Sheraliev, BM	Fergana	Syr Darya	Local fish market			28/01/2018
603.	Ctenopharyngodon_idella_111	SWU28012018111	FFU539-21	MW649729	Xenocyprididae	*Ctenopharyngodon idella*	Sheraliev, BM	Sheraliev, BM	Fergana	Syr Darya	Local fish market			28/01/2018
604.	Ctenopharyngodon_idella_168	SWU17072018168	FFU540-21	MW649730	Xenocyprididae	*Ctenopharyngodon idella*	Sheraliev, BM	Sheraliev, BM	Bukhara	Zeravshan	Dzhalvan canal	40.149666	64.885400	17/07/2018
605.	Ctenopharyngodon_idella_466	SWU28072019466	FFU541-21	MW649731	Xenocyprididae	*Ctenopharyngodon idella*	Sheraliev, BM	Sheraliev, BM	Bukhara	Zeravshan	Zeravshan	40.071012	64.778429	28/07/2019
606.	Ctenopharyngodon_idella_672	SWU21082019672	FFU542-21	MW649732	Xenocyprididae	*Ctenopharyngodon idella*	Rozimov, A	Sheraliev, BM	Khorazm	Amu Darya	Amu Darya	41.880305	60.450888	21/08/2019
607.	Hemiculter_leucisculus_019	SWU16082016019	FFU543-21	MW649733	Xenocyprididae	*Hemiculter leucisculus*	Sheraliev, BM	Sheraliev, BM	Qashqadaryo	Amu Darya	Qorasuv Stream	38.570458	66.296166	16/08/2016
608.	Hemiculter_leucisculus_020	SWU16082016020	FFU544-21	MW649734	Xenocyprididae	*Hemiculter leucisculus*	Sheraliev, BM	Sheraliev, BM	Qashqadaryo	Amu Darya	Qorasuv Stream	38.570458	66.296166	16/08/2016
609.	Hemiculter_leucisculus_126	SWU08022018126	FFU545-21	MW649735	Xenocyprididae	*Hemiculter leucisculus*	Sheraliev, BM	Sheraliev, BM	Fergana	Syr Darya	Sokh River	40.340176	71.013559	08/02/2018
610.	Hemiculter_leucisculus_151	SWU16072018151	FFU546-21	MW649736	Xenocyprididae	*Hemiculter leucisculus*	Sheraliev, BM	Sheraliev, BM	Bukhara	Zeravshan	Zeravshan	40.146944	64.891666	16/07/2018
611.	Hemiculter_leucisculus_152	SWU16072018152	FFU547-21	MW649737	Xenocyprididae	*Hemiculter leucisculus*	Sheraliev, BM	Sheraliev, BM	Bukhara	Zeravshan	Zeravshan	40.146944	64.891666	16/07/2018
612.	Hemiculter_leucisculus_166	SWU17072018166	FFU548-21	MW649738	Xenocyprididae	*Hemiculter leucisculus*	Sheraliev, BM	Sheraliev, BM	Bukhara	Zeravshan	Dzhalvan canal	40.149666	64.885400	17/07/2018
613.	Hemiculter_leucisculus_167	SWU17072018167	FFU549-21	MW649739	Xenocyprididae	*Hemiculter leucisculus*	Sheraliev, BM	Sheraliev, BM	Bukhara	Zeravshan	Dzhalvan canal	40.149666	64.885400	17/07/2018
614.	Hemiculter_leucisculus_233	SWU16082016233	FFU550-21	MW649740	Xenocyprididae	*Hemiculter leucisculus*	Sheraliev, BM	Sheraliev, BM	Qashqadaryo	Amu Darya	Qorasuv Stream	38.570458	66.296166	16/08/2016
615.	Hemiculter_leucisculus_318	SWU21072019318	FFU551-21	MW649741	Xenocyprididae	*Hemiculter leucisculus*	Sheraliev, BM	Sheraliev, BM	Surxondaryo	Amu Darya	Surkhan Darya	37.238888	67.329722	21/07/2019
616.	Hemiculter_leucisculus_319	SWU21072019319	FFU552-21	MW649742	Xenocyprididae	*Hemiculter leucisculus*	Sheraliev, BM	Sheraliev, BM	Surxondaryo	Amu Darya	Surkhan Darya	37.238888	67.329722	21/07/2019
617.	Hemiculter_leucisculus_324	SWU21072019324	FFU553-21	MW649743	Xenocyprididae	*Hemiculter leucisculus*	Sheraliev, BM	Sheraliev, BM	Surxondaryo	Amu Darya	Surkhan Darya	37.238888	67.329722	21/07/2019
618.	Hemiculter_leucisculus_391	SWU24072019391	FFU554-21	MW649744	Xenocyprididae	*Hemiculter leucisculus*	Sheraliev, BM	Sheraliev, BM	Surxondaryo	Amu Darya	Surkhan Darya	37.823333	67.618611	24/07/2019
619.	Hemiculter_leucisculus_392	SWU24072019392	FFU555-21	MW649745	Xenocyprididae	*Hemiculter leucisculus*	Sheraliev, BM	Sheraliev, BM	Surxondaryo	Amu Darya	Surkhan Darya	37.823333	67.618611	24/07/2019
620.	Hemiculter_leucisculus_401	SWU24072019401	FFU556-21	MW649746	Xenocyprididae	*Hemiculter leucisculus*	Sheraliev, BM	Sheraliev, BM	Surxondaryo	Amu Darya	Surkhan Darya	37.823333	67.618611	24/07/2019
621.	Hemiculter_leucisculus_453	SWU28072019453	FFU557-21	MW649747	Xenocyprididae	*Hemiculter leucisculus*	Sheraliev, BM	Sheraliev, BM	Bukhara	Zeravshan	Zeravshan	40.071012	64.778429	28/07/2019
622.	Hemiculter_leucisculus_454	SWU28072019454	FFU558-21	MW649748	Xenocyprididae	*Hemiculter leucisculus*	Sheraliev, BM	Sheraliev, BM	Bukhara	Zeravshan	Zeravshan	40.071012	64.778429	28/07/2019
623.	Hemiculter_leucisculus_455	SWU28072019455	FFU559-21	MW649749	Xenocyprididae	*Hemiculter leucisculus*	Sheraliev, BM	Sheraliev, BM	Bukhara	Zeravshan	Zeravshan	40.071012	64.778429	28/07/2019
624.	Hemiculter_leucisculus_456	SWU28072019456	FFU560-21	MW649750	Xenocyprididae	*Hemiculter leucisculus*	Sheraliev, BM	Sheraliev, BM	Bukhara	Zeravshan	Zeravshan	40.071012	64.778429	28/07/2019
625.	Hemiculter_leucisculus_469	SWU29072019469	FFU561-21	MW649751	Xenocyprididae	*Hemiculter leucisculus*	Sheraliev, BM	Sheraliev, BM	Bukhara	Zeravshan	Unnamed stream	40.147222	64.905805	29/07/2019
626.	Hemiculter_leucisculus_470	SWU29072019470	FFU562-21	MW649752	Xenocyprididae	*Hemiculter leucisculus*	Sheraliev, BM	Sheraliev, BM	Bukhara	Zeravshan	Unnamed stream	40.147222	64.905805	29/07/2019
627.	Hemiculter_leucisculus_478	SWU30072019478	FFU563-21	MW649753	Xenocyprididae	*Hemiculter leucisculus*	Sheraliev, BM	Sheraliev, BM	Khorazm	Amu Darya	Unnamed canal	41.282666	60.803916	30/07/2019
628.	Hemiculter_leucisculus_479	SWU30072019479	FFU564-21	MW649754	Xenocyprididae	*Hemiculter leucisculus*	Sheraliev, BM	Sheraliev, BM	Khorazm	Amu Darya	Unnamed canal	41.282666	60.803916	30/07/2019
629.	Hemiculter_leucisculus_480	SWU30072019480	FFU565-21	MW649755	Xenocyprididae	*Hemiculter leucisculus*	Sheraliev, BM	Sheraliev, BM	Khorazm	Amu Darya	Unnamed canal	41.282666	60.803916	30/07/2019
630.	Hemiculter_leucisculus_696	SWU27042019696	FFU566-21	MW649756	Xenocyprididae	*Hemiculter leucisculus*	Allayarov, S	Sheraliev, BM	Surxondaryo	Amu Darya	Karatag river	38.363055	68.072059	27/04/2019
631.	Hemiculter_leucisculus_711	SWU19042020711	FFU567-21	MW649757	Xenocyprididae	*Hemiculter leucisculus*	Rozimov, A	Sheraliev, BM	Khorazm	Amu Darya	Unnamed stream	41.540222	60.166666	19/04/2020
632.	Hypophthalmichthys_molitrix_089	SWU23012018089	FFU568-21	MW649758	Xenocyprididae	*Hypophthalmichthys molitrix*	Sheraliev, BM	Sheraliev, BM	Fergana	Syr Darya	Local fish market			23/01/2018
633.	Hypophthalmichthys_molitrix_090	SWU23012018090	FFU569-21	MW649759	Xenocyprididae	*Hypophthalmichthys molitrix*	Sheraliev, BM	Sheraliev, BM	Fergana	Syr Darya	Local fish market			23/01/2018
634.	Hypophthalmichthys_molitrix_091	SWU23012018091	FFU570-21	MW649760	Xenocyprididae	*Hypophthalmichthys molitrix*	Sheraliev, BM	Sheraliev, BM	Fergana	Syr Darya	Local fish market			23/01/2018
635.	Hypophthalmichthys_molitrix_097	SWU25012018097	FFU571-21	MW649761	Xenocyprididae	*Hypophthalmichthys molitrix*	Sheraliev, BM	Sheraliev, BM	Fergana	Syr Darya	Fergana Canal	40.388497	71.327960	25/01/2018
636.	Hypophthalmichthys_molitrix_182	SWU18072018182	FFU572-21	MW649762	Xenocyprididae	*Hypophthalmichthys molitrix*	Sheraliev, BM	Sheraliev, BM	Navoiy	Zeravshan	Tudakul Lake	39.801388	64.772222	18/07/2018
637.	Hypophthalmichthys_molitrix_450	SWU28072019450	FFU573-21	MW649763	Xenocyprididae	*Hypophthalmichthys molitrix*	Sheraliev, BM	Sheraliev, BM	Bukhara	Zeravshan	Zeravshan	40.071012	64.778429	28/07/2019
638.	Hypophthalmichthys_molitrix_451	SWU28072019451	FFU574-21	MW649764	Xenocyprididae	*Hypophthalmichthys molitrix*	Sheraliev, BM	Sheraliev, BM	Bukhara	Zeravshan	Zeravshan	40.071012	64.778429	28/07/2019
639.	Hypophthalmichthys_molitrix_452	SWU28072019452	FFU575-21	MW649765	Xenocyprididae	*Hypophthalmichthys molitrix*	Sheraliev, BM	Sheraliev, BM	Bukhara	Zeravshan	Zeravshan	40.071012	64.778429	28/07/2019
640.	Hypophthalmichthys_nobilis_112	SWU29012018112	FFU576-21	MW649766	Xenocyprididae	*Hypophthalmichthys nobilis*	Sheraliev, BM	Sheraliev, BM	Fergana	Syr Darya	Local fish market			29/01/2018
641.	Hypophthalmichthys_nobilis_113	SWU29012018113	FFU577-21	MW649767	Xenocyprididae	*Hypophthalmichthys nobilis*	Sheraliev, BM	Sheraliev, BM	Fergana	Syr Darya	Local fish market			29/01/2018
642.	Hypophthalmichthys_nobilis_114	SWU29012018114	FFU578-21	MW649768	Xenocyprididae	*Hypophthalmichthys nobilis*	Sheraliev, BM	Sheraliev, BM	Fergana	Syr Darya	Local fish market			29/01/2018
643.	Hypophthalmichthys_nobilis_115	SWU29012018115	FFU579-21	MW649769	Xenocyprididae	*Hypophthalmichthys nobilis*	Sheraliev, BM	Sheraliev, BM	Fergana	Syr Darya	Local fish market			29/01/2018
644.	Hypophthalmichthys_nobilis_116	SWU29012018116	FFU580-21	MW649770	Xenocyprididae	*Hypophthalmichthys nobilis*	Sheraliev, BM	Sheraliev, BM	Fergana	Syr Darya	Local fish market			29/01/2018
645.	Mylopharyngodon_piceus_714	SWU29052020714	FFU581-21	MW649771	Xenocyprididae	*Mylopharyngodon piceus*	Rozimov, A	Sheraliev, BM	Khorazm	Amu Darya	Amu Darya	41.550111	60.868916	29/05/2020
646.	Mylopharyngodon_piceus_715	SWU06062020715	FFU582-21	MW649772	Xenocyprididae	*Mylopharyngodon piceus*	Rozimov, A	Sheraliev, BM	Khorazm	Amu Darya	Amu Darya	41.550111	60.868916	06/06/2020
647.	Opsariichthys_cf_bidens_027	SWU31082016027	FFU583-21	MW649773	Xenocyprididae	*Opsariichthys bidens*	Sheraliev, BM	Sheraliev, BM	Namangan	Syr Darya	Syr Darya	40.882777	71.442222	31/08/2016
648.	Opsariichthys_cf_bidens_028	SWU31082016028	FFU584-21	MW649774	Xenocyprididae	*Opsariichthys bidens*	Sheraliev, BM	Sheraliev, BM	Namangan	Syr Darya	Syr Darya	40.882777	71.442222	31/08/2016
649.	Opsariichthys_cf_bidens_029	SWU31082016029	FFU585-21	MW649775	Xenocyprididae	*Opsariichthys bidens*	Sheraliev, BM	Sheraliev, BM	Namangan	Syr Darya	Syr Darya	40.882777	71.442222	31/08/2016
650.	Opsariichthys_cf_bidens_030	SWU31082016030	FFU586-21	MW649776	Xenocyprididae	*Opsariichthys bidens*	Sheraliev, BM	Sheraliev, BM	Namangan	Syr Darya	Syr Darya	40.882777	71.442222	31/08/2016
651.	Opsariichthys_cf_bidens_032	SWU31082016032	FFU587-21	MW649777	Xenocyprididae	*Opsariichthys bidens*	Sheraliev, BM	Sheraliev, BM	Namangan	Syr Darya	Syr Darya	40.882777	71.442222	31/08/2016
652.	Opsariichthys_cf_bidens_389	SWU24072019389	FFU588-21	MW649778	Xenocyprididae	*Opsariichthys bidens*	Sheraliev, BM	Sheraliev, BM	Surxondaryo	Amu Darya	Surkhan Darya	37.823333	67.618611	24/07/2019
653.	Opsariichthys_cf_bidens_608	SWU18082019608	FFU589-21	MW649779	Xenocyprididae	*Opsariichthys bidens*	Sheraliev, BM	Sheraliev, BM	Tashkent	Syr Darya	Chirchik River	41.274444	69.389166	18/08/2019
654.	Parabramis_pekinensis_131	SWU20022018131	FFU590-21	MW649780	Xenocyprididae	*Parabramis pekinensis*	Sheraliev, BM	Sheraliev, BM	Navoiy	Zeravshan	Tudakul Lake	39.907075	64.860898	20/02/2018
655.	Parabramis_pekinensis_132	SWU20022018132	FFU591-21	MW649781	Xenocyprididae	*Parabramis pekinensis*	Sheraliev, BM	Sheraliev, BM	Navoiy	Zeravshan	Tudakul Lake	39.907075	64.860898	20/02/2018
656.	Parabramis_pekinensis_150	SWU16072018150	FFU592-21	MW649782	Xenocyprididae	*Parabramis pekinensis*	Sheraliev, BM	Sheraliev, BM	Bukhara	Zeravshan	Zeravshan	40.146944	64.891666	16/07/2018
657.	Parabramis_pekinensis_165	SWU17072018165	FFU593-21	MW649783	Xenocyprididae	*Parabramis pekinensis*	Sheraliev, BM	Sheraliev, BM	Bukhara	Zeravshan	Dzhalvan canal	40.149666	64.885400	17/07/2018
658.	Parabramis_pekinensis_173	SWU18082018173	FFU594-21	MW649784	Xenocyprididae	*Parabramis pekinensis*	Sheraliev, BM	Sheraliev, BM	Navoiy	Zeravshan	Tudakul Lake	39.801388	64.772222	18/07/2018
659.	Parabramis_pekinensis_174	SWU18082018174	FFU595-21	MW649785	Xenocyprididae	*Parabramis pekinensis*	Sheraliev, BM	Sheraliev, BM	Navoiy	Zeravshan	Tudakul Lake	39.801388	64.772222	18/07/2018
660.	Parabramis_pekinensis_175	SWU18082018175	FFU596-21	MW649786	Xenocyprididae	*Parabramis pekinensis*	Sheraliev, BM	Sheraliev, BM	Navoiy	Zeravshan	Tudakul Lake	39.801388	64.772222	18/07/2018
661.	Parabramis_pekinensis_178	SWU18082018178	FFU597-21	MW649787	Xenocyprididae	*Parabramis pekinensis*	Sheraliev, BM	Sheraliev, BM	Navoiy	Zeravshan	Tudakul Lake	39.801388	64.772222	18/07/2018
662.	Parabramis_pekinensis_179	SWU18082018179	FFU598-21	MW649788	Xenocyprididae	*Parabramis pekinensis*	Sheraliev, BM	Sheraliev, BM	Navoiy	Zeravshan	Tudakul Lake	39.801388	64.772222	18/07/2018
663.	Parabramis_pekinensis_457	SWU28072019457	FFU599-21	MW649789	Xenocyprididae	*Parabramis pekinensis*	Sheraliev, BM	Sheraliev, BM	Bukhara	Zeravshan	Zeravshan	40.071012	64.778429	28/07/2019
664.	Parabramis_pekinensis_458	SWU28072019458	FFU600-21	MW649790	Xenocyprididae	*Parabramis pekinensis*	Sheraliev, BM	Sheraliev, BM	Bukhara	Zeravshan	Zeravshan	40.071012	64.778429	28/07/2019
665.	Parabramis_pekinensis_481	SWU30072019481	FFU601-21	MW649791	Xenocyprididae	*Parabramis pekinensis*	Sheraliev, BM	Sheraliev, BM	Khorazm	Amu Darya	Unnamed canal	41.282666	60.803916	30/07/2019
666.	Parabramis_pekinensis_482	SWU30072019482	FFU602-21	MW649792	Xenocyprididae	*Parabramis pekinensis*	Sheraliev, BM	Sheraliev, BM	Khorazm	Amu Darya	Unnamed canal	41.282666	60.803916	30/07/2019

TABLE S2. Fish species identification from GenBank and BOLD databases

No	Species name	GenBank		BOLD	
		Species name	Identity (%)	Species name	Identity (%)
1.	*Rhodeus ocellatus*	*Rhodeus ocellatus*	100	*Rhodeus ocellatus*	100
2.	*Rhodeus* sp.	***Rhodeus ocellatus***	**92.44**	**No match**	**0**
3.	*Acipenser baerii*	*Acipenser baerii*	99.71-99.85	*Acipenser baerii*	100
4.	*Pseudoscaphirhynchus hermanni*	***Pseudoscaphirhynchus kaufmanni***	**99.71**	***Pseudoscaphirhynchus kaufmanni***	**99.85-100**
5.	*Pseudoscaphirhynchus kaufmanni*	*Pseudoscaphirhynchus kaufmanni*	100	*Pseudoscaphirhynchus kaufmanni*	99.85-100
6.	*Channa argus*	*Channa argus*	100	*Channa argus*	100
7.	*Sabanejewia aurata*	*Sabanejewia aurata*	98.58-98.74	*Sabanejewia aurata*	98.77-99.23
8.	*Cottus spinulosus*	***Cottus ricei***	**98.47**	***Cottus ricei***	**98.48**
9.	*Capoeta heratensis*	*Capoeta capoeta heratensis*	100	*Capoeta heratensis*	100
10.	*Carassius auratus*	*Carassius auratus*	100	*Carassius auratus*	100
11.	*Carassius gibelio*	*Carassius gibelio*	100	*Carassius gibelio*	100
12.	*Cyprinus carpio*	*Cyprinus carpio*	100	*Cyprinus carpio*	100
13.	*Luciobarbus brachycephalus*	*Luciobarbus brachycephalus*	99.70	*Luciobarbus brachycephalus*	99.54-99.69
14.	*Luciobarbus conocephalus*	***Luciobarbus capito***	**98.83-100**	*Luciobarbus conocephalus*	99.85-100
15.	*Schizothorax eurystomus*	*Schizothorax eurystomus*	100	*Schizothorax eurystomus*	100
16.	*Schizothorax fedtschenkoi*	***Schizothorax davidi***	**95.29**	**No match**	**0**
17.	*Schizothorax* sp.	***Schizothorax biddulphi***	**95.31**	**No match**	**0**
18.	*Esox lucius*	*Esox lucius*	99.55	*Esox lucius*	99.55
19.	*Neogobius melanostomus*	*Neogobius melanostomus*	100	*Neogobius melanostomus*	100
20.	*Neogobius pallasi*	***Neogobius fluviatilis***	**93.42**	*Neogobius pallasi*	99.38
21.	*Rhinogobius* sp.	***Rhinogobius sp***	**97.18**	*Rhinogobius sp.*	100
22.	*Abbottina rivularis*	*Abbottina rivularis*	99.71	*Abbottina rivularis*	99.71
23.	*Gobio lepidolaemus*	*Gobio lepidolaemus*	100	*Gobio lepidolaemus*	100
24.	*Gobio nigrescens*	*Gobio nigrescens*	100	*Gobio nigrescens*	99.54
25.	*Gobio sibiricus*	*Gobio sibiricus*	99.54-100	*Gobio sibiricus*	99.54
26.	*Pseudorasbora parva*	*Pseudorasbora parva*	100	*Pseudorasbora parva*	100
27.	*Abramis brama*	*Abramis brama*	100	*Abramis brama*	100
28.	*Alburnoides holciki*	*Alburnoides holciki*	100	*Alburnoides holciki*	100
29.	*Alburnus taeniatus*	***Alburnus escherichii***	**98.82**	***Alburnus escherichii***	**98.92**
30.	*Alburnus chalcoides*	*Alburnus chalcoides*	99.85	*Alburnus chalcoides*	100
31.	*Alburnus oblongus*	***Alburnus escherichii***	**98.39**	***Alburnus escherichii***	**98.62**
32.	*Capoetobrama kuschakewitschi*	***Vimba melanops***	**96.47**	**No match**	**0**
33.	*Leuciscus aspius*	*Leuciscus aspius*	99.70	*Leuciscus aspius*	99.85
34.	*Leuciscus lehmanni*	***Leuciscus baicalensis***	**99.85**	***Leuciscus baicalensis***	**100**
35.	*Petroleuciscus squaliusculus*	***Leuciscus baicalensis***	**99.71**	***Leuciscus baicalensis***	**99.85**
36.	*Pelecus cultratus*	*Pelecus cultratus*	100	*Pelecus cultratus*	100
37.	*Rutilus lacustris*	*Rutilus lacustris*	100	*Rutilus lacustris*	100
38.	*Dzihunia amudarjensis*	***Schistura longa***	**86.66**	**No match**	**0**
39.	*Dzihunia* sp.1	***Schistura reticulofasciata***	**87.85**	**No match**	**0**
40.	*Dzihunia* sp. 2	***Schistura longa***	**87.98**	**No match**	**0**
41.	*Dzihunia* sp. 3	***Schistura longa***	**88.42**	**No match**	**0**
42.	*Paracobitis longicauda*	***Paracobitis persa***	**94.02**	**No match**	**0**

43.	*Triplopysa ferganaensis*	***Triplophysa orientalis***	**94.69**	**No match**	**0**
44.	*Triplophysa strauchii*	*Triplophysa strauchii*	99.27	*Triplophysa strauchii*	99.12
45.	*Triplohysa* sp.1	***Triplophysa aliensis***	**98.37**	***Triplophysa aliensis***	**98.36**
46.	*Triplopysa* sp. 2	***Triplophysa scleroptera***	**95.43**	**No match**	**0**
47.	*Sander lucioperca*	*Sander lucioperca*	99.85	*Sander lucioperca*	100
48.	*Gambusia holbrooki*	*Gambusia holbrooki*	99.85-100	*Gambusia holbrooki*	100
49.	*Oncorhynchus mykiss*	*Oncorhynchus mykiss*	99.85	*Oncorhynchus mykiss*	100
50.	*Silurus glanis*	*Silurus glanis*	99.27-99.67	*Silurus glanis*	99.69
51.	*Glyptosternon oschanini*	*Glyptosternon oschanini*	99.84-100	**No match**	**0**
52.	*Glyptosternon* sp.	***Glyptosternon oschanini***	**97.74-97.90**	**No match**	**0**
53.	*Ctenopharyngodon idella*	*Ctenopharyngodon idella*	100	*Ctenopharyngodon idella*	100
54.	*Hemiculter leuciscuslus*	*Hemiculter leuciscuslus*	99.85	*Hemiculter leucisculus*	99.85
55.	*Hypophthalmichthys molitrix*	*Hypophthalmichthys molitrix*	99.56-100	*Hypophthalmichthys molitrix*	100
56.	*Hypophthalmichthys nobilis*	*Hypophthalmichthys nobilis*	100	*Hypophthalmichthys nobilis*	100
57.	*Mylopharyngodon piceus*	*Mylopharyngodon piceus*	99.85	*Mylopharyngodon piceus*	100
58.	*Opsariichthys bidens*	*Opsariichthys bidens*	98.97-100	*Opsariichthys bidens*	100
59.	*Parabramis pekinensis*	*Parabramis pekinensis*	99.85	*Parabramis pekinensis*	100

TABLE S3. Barcode Index Number (BIN), average and maximum intraspecific distance and distance to nearest neighbor (NN).

No.	Species name	BINs	Average intraspecific distance (%)	Maximum intraspecific distance (%)	Nearest neighbor (Process ID)	Distance to nearest neighbor (%)
1.	*Rhodeus ocellatus*	BOLD:ADC7506	0.08	0.15	*Rhodeus* sp. (FFU022-20)	9.78
2.	*Rhodeus* sp.	BOLD:AEH1279	N/A	0.00	*Rhodeus ocellatus* (FFU018-20)	9.78
3.	*Acipenser baerii*	BOLD:AAA3850	0.00	0.00	*Pseudoscaphirhynchus kaufmanni* (FFU029-20)	5.07
4.	*Pseudoscaphirhynchus hermanni*	BOLD:ABY4771	0.00	0.00	*Pseudoscaphirhynchus kaufmanni* (FFU029-20)	0.00
5.	*Pseudoscaphirhynchus kaufmanni*		0.49	0.74	*Pseudoscaphirhynchus hermanni* (FFU026-20)	0.00
6.	*Channa argus*	BOLD:ABW0047	0.05	0.15	*Silurus glanis* (FFU529-21)	21.21
7.	*Sabanejewia aurata*	BOLD:ADL3084	0.26	1.04	*Paracobitis longicauda* (FFU427-21)	18.07
8.	*Cottus spinulosus*	BOLD:ABX6144	0.18	0.44	*Rhinogobius* sp. (FFU210-210-21)	17.76
9.	*Capoeta heratensis*	BOLD:ABV5006	0.26	0.74	*Luciobarbus conocephalus* (FFU159-21)	5.73
10.	*Carassius auratus*	BOLD:AAA7176	0.25	0.59	*Carassius gibelio* (FFU116-20)	0.00
11.	*Carassius gibelio*		0.32	0.89	*Carassius auratus* (FFU096-20)	0.00
12.	*Cyprinus carpio*	BOLD:AAA7175	0.07	0.29	*Schizothorax fedtschenkoi* (FFU203-21)	10.38
13.	*Luciobarbus brachycephalus*	BOLD:ADC7698	0.25	0.44	*Luciobarbus conocephalus* (FFU159-21)	2.40
14.	*Luciobarbus conocephalus*	BOLD:AEH2022	0.08	0.15	*Luciobarbus brachycephalus* (FFU141-20)	2.40
15.	*Schizothorax eurystomus*	BOLD:AAG2555	0.24	0.59	*Schizothorax* sp. (FFU666-21)	5.73
16.	*Schizothorax fedtschenkoi*	BOLD:AEH2828	0.09	0.15	*Schizothorax* sp. (FFU666-21)	2.71
17.	*Schizothorax* sp.	BOLD:AEH5960	0.35	0.59	*Schizothorax fedtschenkoi* (FFU203-21)	2.71
18.	*Esox lucius*	BOLD:AAA5988	0.00	0.00	*Paracobitis longicauda* (FFU441-21)	20.65
19.	*Neogobius melanostomus*	BOLD:AAC0218	N/A	0.00	*Neogobius pallasi* (FFU207-21)	11.78
20.	*Neogobius pallasi*	BOLD:ADL4983	N/A	0.00	*Neogobius melanostomus* (FFU207-21)	11.78
21.	*Rhinogobius* sp.	BOLD:ACB4145	0.00	0.00	*Cottus spinulosus* (FFU066-20)	17.76
22.	*Abbottina rivularis*	BOLD:ACM1972	0.16	0.44	*Petroleuciscus squaliusculus* (FFU394-21)	15.89
23.	*Gobio nigrescens*	BOLD:ADM0400	0.00	0.00	*Gobio sibiricus* (FFU283-21)	5.55
24.	*Gobio lepidolaemus*	BOLD:AAC5606	0.28	0.59	*Gobio sibiricus* (FFU283-21)	0.89
25.	*Gobio sibiricus*		0.00	0.00	*Gobio lepidolaemus* (FFU271-21)	0.89
26.	*Pseudorasbora parva*	BOLD:AAD0138	0.63	1.48	*Gobio lepidolaemus* (FFU271-21)	15.33
27.	*Abramis brama*	BOLD:AAC8592	0.00	0.00	*Capoetobrama kuschakewitschi* (FFU365-21)	6.37
28.	*Alburnoides holciki*	BOLD:ADK0904	0.36	1.19	*Abramis brama*(FFU296-21)	10.37
29.	*Alburnus chalcoides*	BOLD:AAB6908	0.13	0.29	*Alburnus taeniatus* (FFU339-21)	3.64
30.	*Alburnus oblongus*	BOLD:AAB6906	0.11	0.29	*Alburnus taeniatus* (FFU339-21)	2.25
31.	*Alburnus taeniatus*		0.00	0.00	*Alburnus oblongus* (FFU361-21)	2.25
32.	*Capoetobrama kuschakewitschi*	BOLD:AEH1599	0.00	0.00	*Abramis brama*(FFU296-21)	6.37
33.	*Leuciscus aspius*	BOLD:AAC8137	N/A	0.00	*Leuciscus lehmanni* (FFU377-21)	6.03
34.	*Leuciscus lehmanni*	BOLD:AAE3495	0.21	0.59	*Petroleuciscus squaliusculus* (FFU391-21)	0.00
35.	*Petroleuciscus squaliusculus*		0.37	0.74	*Leuciscus lehmanni* (FFU380-21)	0.00
36.	*Pelecus cultratus*	BOLD:AAF5575	0.07	0.15	*Abramis brama*(FFU296-21)	10.04
37.	*Rutilus lacustris*	BOLD:AAA5492	0.21	0.44	*Capoetobrama kuschakewitschi* (FFU365-21)	8.02
38.	*Dzihunia amudarjensis*	BOLD:AEH1522	0.00	0.00	*Dzihunia* sp.1 (FFU603-21)	7.16
39.	*Dzihunia* sp.1	BOLD:AEH3205	0.16	0.44	*Dzihunia* sp.2 (FFU643-21)	5.05
40.	*Dzihunia* sp. 2	BOLD:AEH3654	0.00	0.00	*Dzihunia* sp.1 (FFU638-21)	5.05
41.	*Dzihunia* sp. 3	BOLD:AEH8655	0.04	0.15	*Dzihunia* sp.1 (FFU603-21)	8.69
42.	*Paracobitis longicauda*	BOLD:AEH0433	0.33	1.18	*Dzihunia* sp.3 (FFU651-21)	13.42
43.	*Triplopysa ferganaensis*	BOLD:AEH3382	0.00	0.00	*Triplophysa* sp.2 (FFU610-21)	5.86
44.	*Triplophysa strauchii*	BOLD:AAH2273	0.22	0.74	*Triplophysa* sp.2 (FFU610-21)	6.54
45.	*Triplohysa* sp.1	BOLD:AEH5574	0.00	0.00	*Triplophysa strauchii* (FFU453-21)	8.32
46.	*Triplopysa* sp. 2	BOLD:AEH6371	0.00	0.00	*Triplophysa ferganaensis* (FFU614-21)	5.86
47.	*Sander lucioperca*	BOLD:AAD1749	0.10	0.29	*Cottus spinulosus* (FFU066-20)	18.79
48.	*Gambusia holbrooki*	BOLD:AAC2757	0.15	0.29	*Rhinogobius* sp. (FFU210-21)	22.19
49.	*Oncorhynchus mykiss*	BOLD:AAA1627	0.15	0.15	*Rhodeus* sp. (FFU022-20)	20.99
50.	*Silurus glanis*	BOLD:ACL1933	0.24	0.59	*Gobio lepidolaemus* (FFU269-21)	18.32
51.	*Glyptosternon oschanini*	BOLD:ADW1603	N/A	0.00	*Glyptosternon* sp. (FFU660-21)	1.94
52.	*Glyptosternon* sp.	BOLD:AEH7649	0.00	0.00	*Glyptosternon oschanini* (FFU-653-21)	1.94
53.	*Ctenopharyngodon idella*	BOLD:ACL1923	0.14	0.29	*Hypophthalmichthys molitrix* (FFU570-21)	9.43

54.	*Hemiculter leuciscuslus*	BOLD:ACB5189	0.44	1.04	*Hypophthalmichthys molitrix* (FFU570-21)	8.37
55.	*Hypophthalmichthys molitrix*	BOLD:AAF6633	0.27	0.74	*Hypophthalmichthys nobilis* (FFU577-21)	3.95
56.	*Hypophthalmichthys nobilis*	BOLD:ADK6840	0.00	0.00	*Hypophthalmichthys molitrix* (FFU572-21)	3.95
57.	*Mylopharyngodon piceus*	BOLD:AAD9723	0.44	0.44	*Parabramis pekinensis* (FFU599-21)	8.29
58.	*Opsariichthys bidens*	BOLD:ADG6045<br>BOLD:ADG6046	0.71	1.49	*Ctenopharyngodon idella* (FFU541-21)	12.35
59.	*Parabramis pekinensis*	BOLD:ACU1740	0.30	0.74	*Mylopharyngodon piceus* (FFU582-21)	8.29
